# Conserved and divergent features of neuronal CaMKII holoenzyme structure, function, and high-order assembly

**DOI:** 10.1016/j.celrep.2021.110168

**Published:** 2021-12-28

**Authors:** Olivia R. Buonarati, Adam P. Miller, Steven J. Coultrap, K. Ulrich Bayer, Steve L. Reichow

**Affiliations:** 1 Department of Pharmacology, University of Colorado Anschutz Medical Campus, Aurora, CO 80045, USA; 2 Department of Chemistry, Portland State University, Portland, OR 97201, USA; 3 These authors contributed equally; 4 Lead contact

## Abstract

Neuronal CaMKII holoenzymes (α and β isoforms) enable molecular signal computation underlying learning and memory but also mediate excitotoxic neuronal death. Here, we provide a comparative analysis of these signaling devices, using single-particle electron microscopy (EM) in combination with biochemical and live-cell imaging studies. In the basal state, both isoforms assemble mainly as 12-mers (but also 14-mers and even 16-mers for the β isoform). CaMKIIα and β isoforms adopt an ensemble of extended activatable states (with average radius of 12.6 versus 16.8 nm, respectively), characterized by multiple transient intra- and inter-holoenzyme interactions associated with distinct functional properties. The extended state of CaMKIIβ allows direct resolution of intra-holoenzyme kinase domain dimers. These dimers could enable cooperative activation by calmodulin, which is observed for both isoforms. High-order CaMKII clustering mediated by inter-holoenzyme kinase domain dimerization is reduced for the β isoform for both basal and excitotoxicity-induced clusters, both *in vitro* and in neurons.

## In brief

The CaMKII holoenzyme enables neuronal signal computation. In a comparative structure-function analysis of the neuronal α and β isoforms, Buonarati et al. find evidence for kinase domain dimers within the holoenzyme that enable a cooperative activation mechanism in both isoforms and inter-holoenzyme interactions that enable high-order aggregate formation under ischemic conditions.

## Graphical Abstract

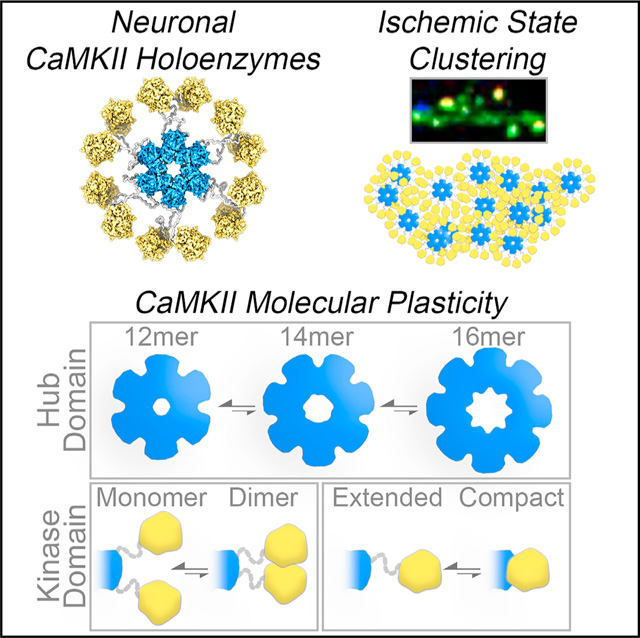

## INTRODUCTION

The Ca^2+^/calmodulin (CaM)-dependent protein kinase II (CaMKII) is a major mediator of long-term plasticity at excitatory glutamatergic synapses in the hippocampus that is required for learning and memory (Bayer and Schulman, 2019; Hell, 2014; Lisman et al., 2012). Beyond these physiological functions, CaMKII also mediates the glutamate excitotoxicity that kills neurons during ischemia (Coultrap et al., 2011; Deng et al., 2017; Vest et al., 2010). Both synaptic plasticity and excitotoxic cell death require the 12-meric CaMKII holoenzyme structure for at least two key regulatory functions: (1) autophosphorylation at T286 (pT286) (Cook et al., 2021; Coultrap et al., 2014; Deng et al., 2017; Giese et al., 1998), which occurs between subunits within a holoenzyme (Hanson et al., 1994) and enables detection of stimulation frequency (De Koninck and Schulman, 1998), and (2) binding to the NMDA-type glutamate receptor subunit GluN2B (Barria and Malinow, 2005; Buonarati et al., 2020; Halt et al., 2012), which also requires the holoenzyme structure (Bayer et al., 2006; Strack et al., 2000) and mediates CaMKII accumulation at synapses during long-term potentiation (LTP) and excitotoxic insults (Bayer et al., 2001; Buonarati et al., 2020; Halt et al., 2012). Both pT286 and GluN2B binding require an initial stimulus by Ca^2+^/CaM but then maintain partial “autonomous” kinase activity even after Ca^2+^/CaM has dissociated (Bayer et al., 2001; Bayer and Schulman, 2019; Miller and Kennedy, 1986). In contrast, the Ca^2+^/CaM-induced clustering of multiple holoenzymes into higher-order aggregates is thought to restrict kinase activity (Hudmon et al., 1996). This aggregation requires ischemia-related conditions (such as low pH and higher ADP than ATP concentration) and mediates the extra-synaptic clustering in response to excitotoxic stimuli (Dosemeci et al., 2000; Hudmon et al., 1996, 2001; Vest et al., 2009) but may contribute also to the synaptic accumulation in response to LTP stimuli (Hudmon et al., 2005).

Together, these holoenzyme functions are thought to provide essential mechanisms for information processing and storage (Bayer and Schulman, 2019; Coultrap and Bayer, 2012b; Rossetti et al., 2017). Thus, elucidating the CaMKII holoenzyme structure that enables these mechanisms has been of long-standing interest, with electron microscopy (EM) studies performed more than 30 years ago (Kanaseki et al., 1991; Woodgett et al., 1983). CaMKII holoenzymes are oligomeric assemblies, with each subunit containing an N-terminal kinase domain, followed by a variable internal linker region that connects to a C-terminal association (or hub) domain that is responsible for oligomerization. More recently, a high-resolution crystal structure showed the 12-meric holoenzyme in a compact conformation, with the N-terminal kinase domains folding back onto the association domain that forms a central hub complex (Chao et al., 2011). Notably, this compact conformation is not activation competent (as the CaM binding regulatory region is buried), and transition between the compact and an extended conformation provided a potential regulatory mechanism for cooperative activation by CaM. However, subsequent studies indicated that the vast majority of kinase domains are in the activation-competent extended conformation (>95%), both *in vitro* and in intact cells (Myers et al., 2017; Sloutsky et al., 2020), indicating that cooperativity must be mediated by a different mechanism.

**Figure 1.**
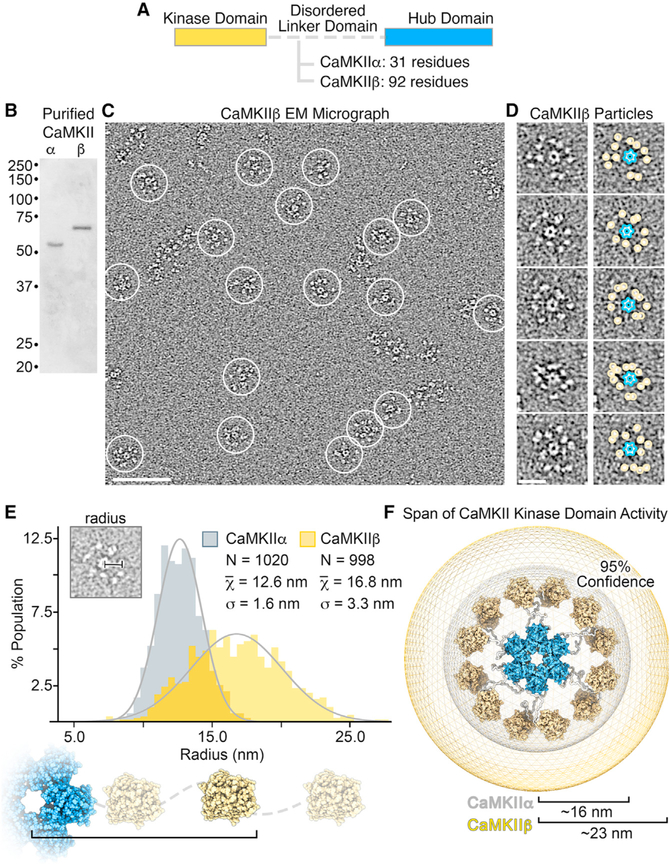
Comparative structural analysis of CaMKII holoenzymes resolved by single-particle EM (A) Diagram showing that the major difference in CaMKIIα and CaMKIIβ domain architecture is within the length of their respective disordered linker domains. (B) SDS-PAGE gel showing that full-length CaMKIIα and CaMKIIβ isoforms purified from S*f*9 cells migrate at the expected molecular weights and display no sign of proteolytic degradation. (C) Electron micrograph of negatively stained CaMKIIβ holoenzymes. Individual complexes are indicated by white circles. Scale bar, 100 nm. (D) Zoomed view of individual particles with the hub complex (blue outline) and resolved kinase domains (yellow circle) indicated. Scale bar, 20 nm. (E) Histogram analysis of kinase radius (center of hub to outer radius of kinase domain) obtained for CaMKIIα (gray; n = 1,020) and CaMKIIβ (yellow; n = 998) particles; bin = 0.5 nm. Gray lines represent a Gaussian fit to the data. Inset: radii were obtained from raw particle images. (F) Schematic illustrating the difference in radial expansion sampled by CaMKIIα (gray) versus CaMKIIβ (yellow), where the outer boundary and values (16 and 23 nm, respectively) represent the 95% confidence interval of measured holoenzyme radii.

To gain deeper insight into the conserved structural and functional features of CaMKII holoenzymes, we performed a comparative single-particle EM analysis to CaMKIIβ, the second most prevalent isoform in neurons (Bayer et al., 1999; Bennett and Kennedy, 1987; Cook et al., 2018; Tombes et al., 2003). As we have previously described for the α isoform (Myers et al., 2017), the association domains of the β isoform form a rigid central hub capable of adopting multiple stoichiometries, whereas their kinase domains were primarily extended away from the hub in an activation-competent and highly flexible manner (see Figure 1). Within the ensemble of conformational states detected by single-particle analysis, our study revealed several intriguing similarities and differences between these isoforms, including formation of higher-order holoenzyme clusters, which are thought to increase in response to ischemic or excitotoxic insults (Hudmon et al., 2001; Vest et al., 2009). In addition, we found direct evidence for dimer formation between kinase domains of the same holoenzyme, a structural feature that can mediate the cooperative activation by CaM found here for both CaMKIIα and β isoforms. Thus, our analysis of CaMKIIβ revealed not only isoform-specific differences, but also generally conserved themes of CaMKII regulation that are facilitated by a dynamic ensemble of multiple transient interactions supported by the holoenzyme architecture.

## RESULTS

### The dodecameric CaMKIIβ holoenzyme adopts an extended kinase radius

We have shown previously that the CaMKIIα holoenzyme adopts a predominant dodecameric (12-mer) assembly, with an extended and flexible activatable-state conformation as visualized by negative stain electron microscopy (NSEM) (Myers et al., 2017). This 12-meric assembly is organized by a central and well-ordered hub domain complex, with 12 kinase domains displayed in an extended fashion via an intrinsically disordered flexible linker domain (Figure 1A). For comparative structural analysis, CaMKIIβ holoenzymes were expressed in eukaryotic cells (S*f*9), purified by chromatographic methods and prepared for NSEM using the same protocols previously described for CaMKIIα. Isolated holoenzymes show no signs of proteolytic degradation by SDS-PAGE (Figure 1B), and CaMKIIβ specimens produced well-resolved assemblies resembling the same “flower-like” appearance of CaMKIIα holoenzyme structures observed by NSEM (Figures 1C and 1D). A defined central ring of density corresponding to the hub domain (~11 nm diameter) was clearly resolved, surrounded by an array of smaller densities, “the petals,” corresponding to tethered kinase domains (~5 nm diameter) (Figures 1C and 1D). However, despite an overall structural similarity, the peripheral kinase domains associated with CaMKIIβ holoenzymes were qualitatively more extended and heterogeneously configured around the hub domain, compared with CaMKIIα holoenzymes. Although the disordered linker domain is not resolvable by NSEM, these differences are consistent with the primary divergence in amino acid sequence between CaMKII isoforms, where in CaMKIIβ the linker domain is ~92 residues long (compared to ~31-residue linker in CaMKIIα) (Cook et al., 2018; Tombes et al., 2003) (Figure 1A).

For both CaMKIIα and β, the flexible linker region facilitates the formation of a variety (or continuum) of conformational states that are not directly amenable to traditional EM image classification and averaging methods. Therefore, for quantitative characterization and comparative analysis between holoenzyme structures, we took advantage of the high contrast of NSEM imaging to conduct a series of measurements and statistical analyses conducted directly on individual holoenzyme particle images obtained from raw micrographs (Myers et al., 2017) (Figures 1D and 1E). In the first set of measurements, the radial extension for each kinase domain (i.e., kinase radius) was obtained by measuring from the center of the hub complex to the center of each kinase domain, and this measurement was then appended by 2.25 nm to account for the approximate radius of the kinase domain (Figures 1E and 1F). For both isoforms, the distribution of kinase domain radii obtained from ~1,000 particle measurements for each isoform appears randomly positioned with apparent Gaussian distribution (Figure 1E). However, the average kinase radius of CaMKIIβ is significantly larger at ~16.8 nm (±0.1 SEM) compared with CaMKIIα assemblies ~12.6 nm (±0.05 SEM) (p < 0.001). As the linker domain is not directly visible by NSEM, the edge-to-edge distance separating the kinase domain and hub domain may be used as an approximation of the linker extension of ~2.7 nm for CaMKIIα and ~6.8 nm for β. These values are consistent with random chain polymer models (traditional random walk model) on the basis of the differences in amino acid lengths of the respective linker domains (Flory, 1975) and further support the idea that CaMKII kinase domains are freely tethered to the central hub domain by intrinsically disordered linker regions. On the basis of these measurements, a 95% confidence of kinase domain positioning can be expected to span a radius of up to ~16 nm for CaMKIIα holoenzymes and up to ~23 nm for β holoenzymes (Figure 1F).

CaMKII holoenzymes can adopt a “compact state” involving kinase-hub domain interaction, but only a minor fraction of CaMKIIα holoenzymes was found in this conformation (Chao et al., 2011; Myers et al., 2017; Sloutsky et al., 2020). From our analysis in Figure 1E, a kinase domain radius measurin less than ~10 nm would potentially place a kinase domain in steric contact with the central hub complex. Consistent with our previous analysis, CaMKIIα holoenzymes showed only a small fraction of individual subunits with kinase domain radii that fall within this category (~3% of kinase domains with radius < 10 nm) (Myers et al., 2017; Figure 1E). In comparison, CaMKIIβ displayed less than 1% of kinase domains with radius < 10 nm observed by NSEM (Figure 1E), indicating that a “compact state” is at most only sparsely populated.

### Autophosphorylation of pT286 in CaMKIIα versus pT287 in CaMKIIβ

The extended radius of the CaMKIIβ compared with the α holoenzyme would lead to a lower local concentration of kinase domains within the space occupied by a holoenzyme, on the basis of simple geometric considerations (~1.0 versus ~2.3 mM; see Figure S1). Thus, we decided to directly compare CaMKIIα versus β purified holoenzymes for autophosphorylation at T286 (in α) or T287 (in β), which occurs as an inter-subunit intra-holoenzyme reaction (Bradshaw et al., 2002; Hanson et al., 1994; Rich and Schulman, 1998). *In vitro* kinase stimulation with Ca^2+^/CaM resulted fast autophosphorylation for both CaMKIIα and β, with no significant differences between T286 and T287 detected throughout the reaction time course (Figure S1). Taken together, these results indicate that the significant difference in the proximity of kinase domains in CaMKIIβ versus CaMKIIα does not significantly affect autophosphorylation kinetics of the holoenzymes.

### CaMKIIβ forms multimeric assemblies of 12- to 16-mers

**Figure 2.**
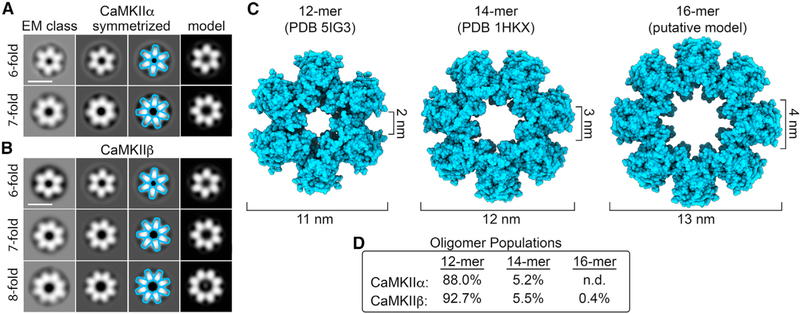
Comparative analysis of CaMKII holoenzyme stoichiometries resolved by single-particle EM (A and B) Single-particle EM image classification and analysis of the central hub domain of CaMKIIα and CaMKIIβ holoenzymes, respectively. Left: focused EM class averages obtained using an applied image mask (15 nm outer diameter) and without applied symmetry. Middle left and right: symmetrized versions of the EM classes as indicated and with resolved features annotated (blue outline). Right: 2D back-projections from atomic models of hub assemblies displayed in (C), filtered to 3.0 nm. Scale bar, 10 nm. (C) Left: atomic model of a dodecameric (12-mer) hub domain (blue surface representation; PDB: 5IG3; Bhattacharyya et al., 2016); center: heptadecameric (14-mer) hub domain (PDB: 1HKX; Hoelz et al., 2003); right: pseudo-atomic model of the putative hexadecameric (16-mer) hub domain resolved in (B). (D) Population of CaMKII holoenzyme stoichiometries resolved by single-particle EM. For the CaMKIIα dataset, n = 10,902, and for the CaMKIIβ dataset, n = 17,347. The 16-mer was not detected (n.d.) in the smaller CaMKIIα image dataset.

Early EM studies had already indicated that CaMKIIα forms mainly 12-mers, but some studies suggested a smaller number of subunits, particularly for the β isoform (Kanaseki et al., 1991; Woodgett et al., 1983). Thus, we decided to further compare the stoichiometry of CaMKIIα versus β holoenzymes, by quantifying the symmetry of their hub domain assemblies. We performed focused two-dimensional (2D) image classification on the NSEM datasets for both isoforms by applying a circular mask to remove signal from the peripheral kinase domains and focus the image alignment procedures on the central hub domain (Figures 2A and 2B). The results of this analysis showed a predominance of particles displaying sixfold radial symmetry, with dimensions and structural features matching 2D projections the dodecameric (12-mer) hub assembly, representing 88% of the population for CaMKIIα and ~92.7% for β (Figures 2A–2D). For both isoforms, a smaller but significant population of hub domains displaying sevenfold radial symmetry were also observed, corresponding to ~5.2% of the population for CaMKIIα and ~5.5% for β holoenzymes (Figures 2A–2D). The dimensions of sevenfold symmetric hubs class averages were also consistent with 2D projections of a previously published crystallographic model of an isolated tetradecameric (14-mer) hub assembly (Hoelz et al., 2003) (Figures 2A–2C).

Remarkably, an additional minor population of hub domain structures was detected with clear eightfold symmetry in the CaMKIIβ image dataset, constituting ~0.4% of the population (Figures 2B–2D). The dimensions of the detected 16-mer hub are ~13 nm in outer diameter, with central pore measuring ~4 nm in diameter (Figure 2B). Pseudo-atomic modeling of hub domain subunits, restricted by the dimensions of the eightfold symmetric projection average, show a reasonable fit, with minimal steric overlap between neighboring subunits, resulting in a putative hexadecameric (16-mer) hub model (Figure 2C). This putative model produces 2D back-projections matching the experimental density (Figure 2B). 16-mer assemblies were not detected in the CaMKIIα dataset. However, given the small population of 16-mers observed for CaMKIIβ, it is possible that this species was simply not detectable by image classification because of the significantly smaller image dataset obtained for CaMKIIα. Nevertheless, inherent differences between isoforms cannot be ruled out.

It is also noteworthy that smaller assemblies (e.g., 8- to 10-mers) were not detected in either dataset. Given the ability of our focused image classification approach to detect populations that represent <1% of species present, it is likely that the CaMKII hub domain is not capable of supporting such configurations under basal-state conditions (at least to any appreciable degree).

### Resolution of kinase domain dimers within intact CaMKIIβ holoenzymes

**Figure 3.**
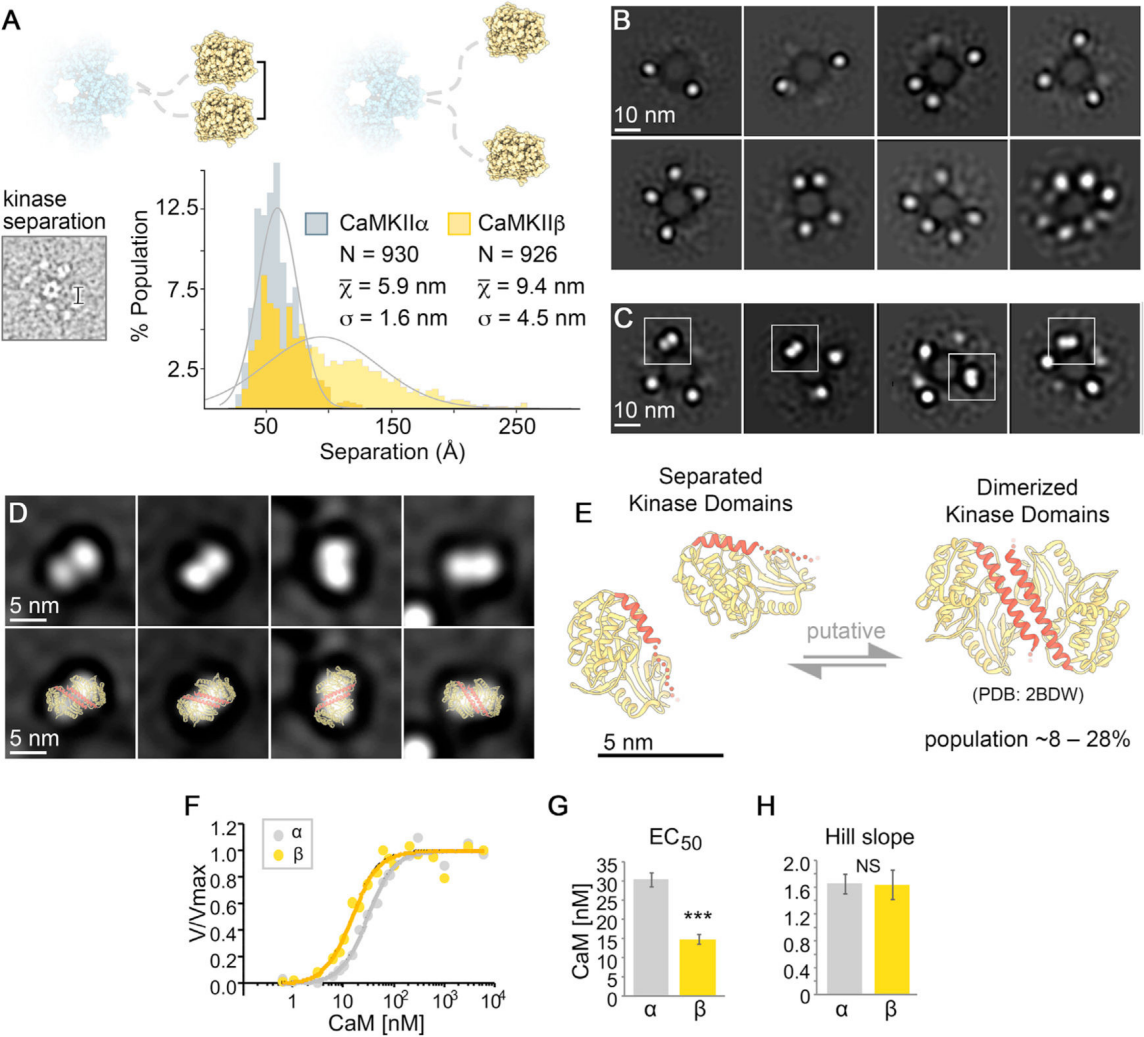
CaMKIIα and CaMKIIβ kinase domains form dimers within the holoenzyme assembly (A) Histogram of measured distance separating neighboring kinase domains (center-to-center) obtained for CaMKIIα (gray; n = 930) and CaMKIIβ (yellow; n = 926) particles; bin = 0.5 nm. Gray lines represent a Gaussian fit to the data. Inset: distances were obtained from raw particle images in EM micrographs. (B and C) Single-particle EM image classification and analysis of CaMKIIβ holoenzymes with an applied mask to remove contribution of the hub domain during the alignment procedure (13.5 nm diameter). In (B), isolated kinase domains appear as punctate densities of ~4–5 nm in diameter. Only a subset of all 12 kinase domains could be resolved in class averages (typically 2 to ~7), because of spatial heterogeneity of kinase domain positions present within the population of CaMKIIβ holoenzymes. (C) Class averages displaying additionally resolved bi-lobed densities with approximate dimensions of ~10 × 5 nm (indicated by white squares). Scale bar, 10 nm in (B) and (C). (D) Top row: zoomed view of bi-lobed densities resolved in class averages in (C); bottom row: with fitted crystallographic structure of the *C. elegans* CaMKIIα kinase/regulatory domain (yellow/red ribbon; PDB: 2BDW; Rosenberg et al., 2005) previously shown to form a dimeric interface involving the regulatory domain (red). (E) Putative model depicting an equilibrium of states involving independent kinase domains (PDB: 2VZ6) (Rellos et al., 2010) and kinase domain dimer (PDB: 2BDW) (Rosenberg et al., 2005) proposed to be present in the context of the CaMKII holoenzyme. The regulatory domain (red) in the dimerized state becomes more ordered and occluded from CaM binding. Scale bar, 5 nm in (D) and (E). The population distribution of kinase domain dimers for CaMKIIβ holoenzymes is presented as 8%–28%, on the basis of separation distances less than 4.5–6.0 nm as being consistent with dimer formation. (F) *In vitro* CaMKII activity in response to varying Ca^2+^/CaM (0.6 nM to 6 μM CaM). The curve fits shown are based on data from two independent experiments (see Figure S2). (G) As expected, CaMKIIβ showed higher sensitivity to CaM activation, with an EC_50_ significantly lower than that of CaMKIIα (14.6 and 30.1 nM, respectively; extra-sum-of-squares F test, ***p < 0.001). (H) Both CaMKIIα and CaMKIIβ exhibited a Hill slope coefficient greater than 1. No differences were observed between isoforms, demonstrating equal CaM cooperativity (1.65 ± 0.15 for α and 1.64 ± 0.22 for β; extra-sum-of-squares F test, p = 0.9668).

Kinase-kinase domain pairing interactions have been proposed as a potential mechanism for CaMKII cooperative activation by Ca^2+^/CaM. Indeed, such dimers have been observed in crystals of the kinase domain (Rosenberg et al., 2005), but they have not yet been directly detected in context of the intact holoenzyme. To assess for the presence of kinase domain dimerization in the context of CaMKII holoenzymes, the separation distance (center to center) between nearest neighbor kinase domains was measured on individual-particle EM images (Figure 3A). For CaMKIIα holoenzymes, the average separation distance was 5.9 nm (±0.05 SEM), with a near Gaussian distribution (Figure 3A, gray). This value is consistent with our previous analysis and with solution-state fluorescence resonance energy transfer (FRET) studies conducted on CaMKIIα holoenzymes (Myers et al., 2017; Thaler et al., 2009) and indicates that the majority of kinase domains are non-interacting (Myers et al., 2017). However, an appreciable fraction of kinase separation distances (~19%) measured less than 4.5 nm, the distance we had previously assigned as the cut-off for potential kinase domain pairing within the context of the holoenzyme (Myers et al., 2017). The cut-off distance was based on the 2–3 nm radius of a kinase domain and represents a compromise in stringency: there is a potential for dimers that appear to exceed this 4.5 nm distance, but not all kinase domain pairs within this distance are likely to represent bona fide dimers. For CaMKIIβ holoenzymes, the average kinase separation distance is significantly larger at 9.4 nm (±0.2 SEM) (Figure 3A, yellow), presumably facilitated by the longer linker domain. Notably, the distribution is skewed from a random Gaussian distribution, toward shorter separation distances, with the most populated distance bin of 4.5–5.0 nm (Figure 3A). The deviation from random distribution toward shorter separation distances may reflect intrinsic interactions between neighboring kinase domains. The fraction of CaMKIIβ kinase domain pairs within 5 nm distance was 15%, the fraction within the maximally possible interaction distance of 6 nm was 28%, and within our original more stringent 4.5 nm cut-off was 8%. Although these data are consistent with the majority of kinase domains adopting a non-interacting configuration, a significant fraction of CaMKIIβ kinase domains are localized within the potential steric contact distance, indicating that kinase domain pairing may represent a significant population of both CaMKIIα and β holoenzyme structures.

To further support this evaluation, we conducted focused 2D image classification on CaMKIIβ kinase domains, this time by masking away the central density of the hub domain prior to image alignment (Figures 3B and 3C). The results of this analysis produced two distinct groups of 2D class averages. The first group appears to resolve isolated densities of ~4–5 nm diameter, corresponding to a subset of the individual kinase domains belonging to a single holoenzyme (Figure 3B). Notably, all 12 kinase domains were not completely resolved in any of these 2D class averages obtained from CaMKIIβ holoenzymes, because of the continuum of kinase domain configurations supported by the extended linker region. In the second group of 2D class averages displayed in Figure 3C, larger elongated densities of ~10 × 5 nm are resolved in addition to the ~4–5 nm individual kinase domain densities. These larger densities have a distinct bi-lobed appearance and dimensions consistent with the crystalized kinase domain dimer structure (Figures 3D and 3E) (Rosenberg et al., 2005). For CaMKIIα, apparent kinase domain dimers were previously resolved in individual-particle images (Myers et al., 2017); however, such structures could not be resolved using 2D classification procedures. We attribute this to limitations associated with local crowding of neighboring kinase domains present in this isoform, which could interfere with image classification. Taken together, these data support the notion that CaMKII kinase domains are capable of forming dimers within the context of the holoenzyme assembly in both CaMKIIα and CaMKIIβ isoforms and may represent ~8%–28% of kinase domains organized by the CaMKII holoenzyme structure (Figure 3E).

### CaMKIIα and β differ in CaM activation constant but not in activation cooperativity

The kinase domain dimers that were found previously in a crystal of the kinase domain (Rosenberg et al., 2005), and potentially here in context of the holoenzyme (see Figure 3), are formed by low-affinity coiled-coil interactions between two regulatory domains; then binding of Ca^2+^/CaM to one regulatory domain would disrupt the interaction and thereby facilitate binding also to the other regulatory domain. This could explain the cooperative activation of CaMKII by CaM (Chao et al., 2011; Myers et al., 2017). To directly compare CaM activation of CaMKIIα versus β purified holoenzymes, we used our established kinase activity assay that measures phosphorylation of the peptide substrate syntide-2 (Coultrap and Bayer, 2011, 2014; Coultrap et al., 2010). Consistent with previous studies (Brocke et al., 1999; De Koninck and Schulman, 1998), CaMKIIβ was more sensitive to Ca^2+^/CaM stimulation than CaMKIIα (half maximal effective concentration [EC_50_] = 15 nM compared with 30 nM; Figures 3F, 3G, and S2). However, the Hill slope was indistinguishable between the isoforms and was determined to be ~1.6 for both (Figures 3H and S2). Such Hill slopes between 1 and 2 are consistent with dimer formation of some but not all kinase domains within a holoenzyme and would indicate that dimer formation is equal between α and β. Indeed, even though the dimer structure was resolved in 2D average classes only for CaMKIIβ but not α (see Figure 3 and Myers et al., 2017), the percentage of kinase domains that are close enough for potential dimer formation are comparable for both α and β isoforms (19% and 8%–28%, respectively) and arguably within the degree of uncertainty on the basis of the limitations of our approach (see discussion).

### CaMKIIα and β form higher-order holoenzyme clusters both *in vitro* and in neurons

**Figure 4.**
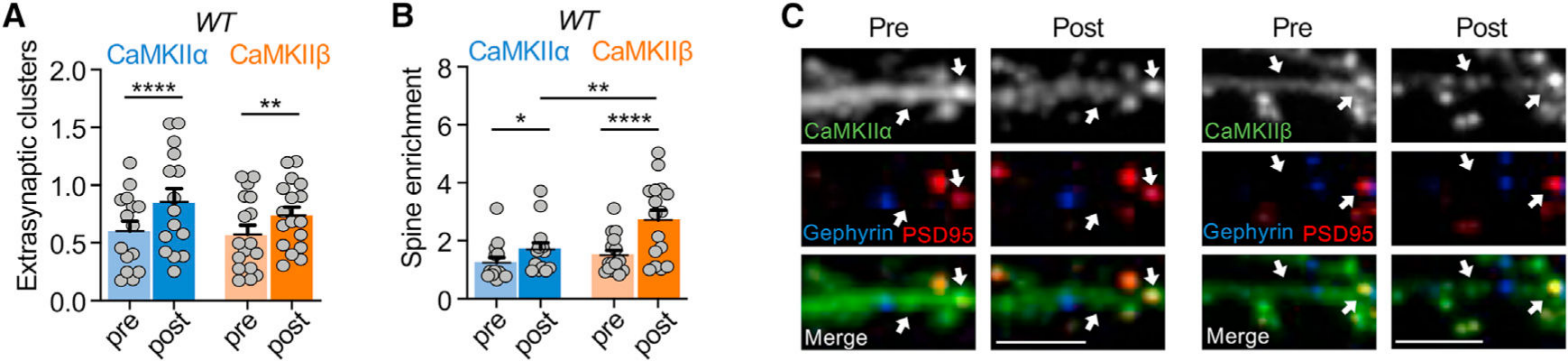
Clustering of CaMKIIα and CaMKIIβ induced by prolonged glutamate in wild-type neurons Quantifications show mean + SEM. *p < 0.05, **p < 0.01, and ****p < 0.0001. Scale bar, 5 μm. (A) Quantification of extra-synaptic clusters induced by excitotoxic glutamate (100 μM glutamate, 5 min) in WT cultured hippocampal neurons (15–17 days *in vitro* [DIV]) (two-way repeated-measures ANOVA, Bonferroni’s test: ****p < 0.0001 for CaMKIIα pre vs. post, n = 15; **p = 0.0049 for CaMKIIβ pre vs. post, n = 17). (B) Quantification of excitatory synapse enrichment induced by excitotoxic glutamate (100 μM glutamate, 5 min) in WT cultured hippocampal neurons (15–17 DIV) (two-way repeated-measures ANOVA, Bonferroni’s test: *p = 0.0457 for CaMKIIα pre vs. post, n = 14; **p < 0.0001 for CaMKIIβ pre vs. post, n = 16; **p = 0.0078 for post CaMKIIα vs. post CaMKIIβ). (C) Representative confocal images show overexpressed YFP-labeled CaMKIIα (left) or CaMKIIβ (right), endogenous PSD95 (in red) to mark excitatory synapses, and endogenous gephyrin (in blue) to mark inhibitory synapses.

Whereas kinase domain dimers within a holoenzyme may contribute to cooperative CaMKII activation, an inter-holoenzyme kinase domain dimer formation is thought to mediate higher-order clustering of CaMKII holoenzymes (although the proposed dimerization mechanisms differ) (Hudmon et al., 2001; Vest et al., 2009). Some clustering can occur basally in neurons and small clusters were also observed on our EM grids, with ~56%–58% of holoenzymes potentially interacting to form holoenzyme pairs and/or higher-order clusters (Figure S3). However, clustering at extra-synaptic sites is majorly enhanced by ischemic conditions (Dosemeci et al., 2000; Hudmon et al., 2005; Vest et al., 2009). Additionally, clustering may contribute to the CaMKII accumulation at excitatory spine synapses during both LTP and ischemia (Hudmon et al., 2005), although CaMKII binding to GluN2B is at least a co-requirement for both (Bayer et al., 2001; Buonarati et al., 2020; Halt et al., 2012). CaMKIIβ has been described to be incompetent for the ischemia-related clustering (at least *in vitro*; Hudmon et al., 2001) but seemed to form at least some basal clusters (see Figure S3). Thus, we decided to directly compare the clustering of CaMKIIα versus β in dissociated hippocampal neurons. For live imaging of synaptic versus extra-synaptic clustering, synapses were identified by expressing intrabodies against the synaptic marker proteins PSD95 and gephyrin, to simultaneously label excitatory and inhibitory synapses, as we have described recently (Buonarati et al., 2020; Cook et al., 2019); the YFP-tagged CaMKII isoforms were expressed to visualize clustering before and after excitotoxic glutamate insults (100 μM for 5 min). CaMKII clusters were detected extra-synaptically, both basally and after stimulation (Figure 4). For both isoforms, excitotoxic stimulation significantly increased extra-synaptic clustering (Figures 4A and 4C) and enrichment at excitatory synapses (Figures 4B and 4C). As previously described for the α isoform (Buonarati et al., 2020), no clustering at inhibitory synapses was observed for CaMKIIβ (Figure S4).

### Homomeric CaMKIIβ holoenzymes show less propensity for higher-order clustering

**Figure 5.**
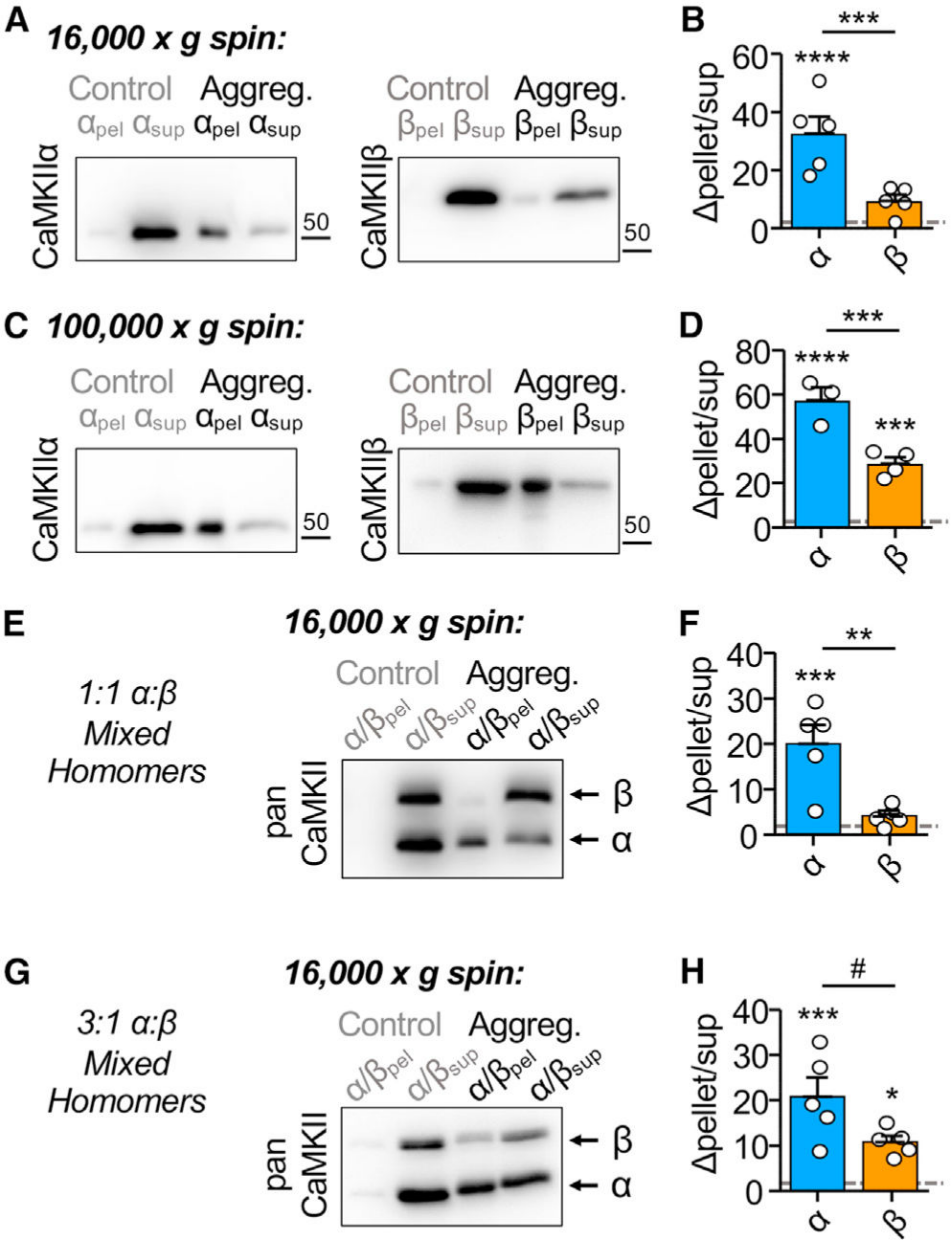
CaMKIIβ self-aggregates *in vitro*, but less than CaMKIIα Quantifications show mean ± SEM. ***p < 0.001. Aggregation was induced by 2 mM Ca^2+^, 1 μM CaM, and 1 μM ADP at low pH (6.4) for 5 min at room temperature. Control samples were incubated in 50 mM EGTA at pH 7.4 and normalized to 1. (A) Representative immunoblots for CaMKIIα and CaMKIIβ. Aggregates were detected in 16,000 × *g* pellets. (B) Quantification of change in pellet enrichment. Only CaMKIIα showed significant clustering under aggregation conditions compared with control (two-way ANOVA, Bonferroni’s test: ****p < 0.0001 for CaMKIIα, p = 0.1577 for CaMKIIβ). CaMKIIβ showed significantly less self-aggregation compared with CaMKIIα (two-way ANOVA, Bonferroni’s test: ***p = 0.0004). (C) Representative immunoblots for CaMKIIα and CaMKIIβ. Aggregates were detected in 100,000 × *g* pellets. (D) Quantification of change in pellet enrichment. Both CaMKIIα and CaMKIIβ showed significant clustering under aggregation conditions, compared with control (two-way ANOVA, Bonferroni’s test: ****p < 0.0001 for CaMKIIα, ***p = 0.0004 for CaMKIIβ). Furthermore, CaMKIIβ shows greater self-aggregation in 100,000 × *g* pellets compared with 16,000 × *g* pellets (two-way ANOVA, Bonferroni’s test: ****p < 0.0001). However, CaMKIIβ still shows significantly lower self-aggregation than CaMKIIα even after 100,000 × *g* (two-way ANOVA, Bonferroni’s test: ***p = 0.0003). (E) Representative immunoblots for 1:1 CaMKIIα/CaMKIIβ mixed homomers. Aggregates were detected in 16,000 × *g* pellets. (F) Quantification of change in pellet enrichment. Only CaMKIIα showed significant clustering under aggregation conditions in 1:1 mixed homomers compared with control (two-way ANOVA, Bonferroni’s test: ***p = 0.0005 for CaMKIIα, p = 0.6800 for CaMKIIβ). CaMKIIβ showed significantly less self-aggregation compared with CaMKIIα (two-way ANOVA, Bonferroni’s test: **p = 0.0100). (G) Representative immunoblots for 1:1 CaMKIIα/CaMKIIβ mixed homomers. Aggregates were detected in 16,000 × *g* pellets. (H) Quantification of change in pellet enrichment. Both CaMKIIα and CaMKIIβ showed significant clustering under aggregation conditions in 3:1 mixed homomers compared with control (two-way ANOVA, Bonferroni’s test: ***p = 0.0005 for CaMKIIα, *p = 0.0283 for CaMKIIβ). No differences in self-aggregation levels were detected between CaMKIIα and CaMKIIβ (two-way ANOVA, Bonferroni’s test: #p = 0.0532).

Somewhat surprisingly, CaMKIIα and β showed the same level of extra-synaptic clustering in hippocampal neurons (Figure 4), even though CaMKIIβ has been described to be incompetent for ischemia-related clustering *in vitro* (Hudmon et al., 2001). Thus, we decided to compare these isoforms also in our *in vitro* clustering assay. Ischemic conditions were mimicked by addition of Ca^2+^/CaM and ADP at a low pH of 6.5; then cluster formation was assessed by differential centrifugation (Vest et al., 2009). Although some amount of both CaMKIIα and β was detected in the 16,000 × *g* pellet under basal conditions, this amount dramatically increased under ischemic conditions only for CaMKIIα but not β (Figures 5A and 5B). The CaMKIIβ isoform appeared to show some minor increase in clustering, but this was not significant. By contrast, in 100,000 × *g* pellets, both isoforms showed a significant increase in precipitation under ischemic conditions (Figures 5C and 5D). Nonetheless, the induced precipitation of CaMKIIβ was significantly less than that of CaMKIIα at either centrifugation speed (Figures 5B and 5D). These results suggest that both isoforms can cluster *in vitro* but that CaMKIIβ forms fewer and/or smaller sized clusters than the α isoform.

Then why was clustering of CaMKIIα and β indistinguishable in wild-type (WT) neurons? One possibility was that CaMKIIβ might efficiently co-cluster with endogenous CaMKIIα. Thus, we decided to test if mixing CaMKIIβ with CaMKIIα can lead to detection of significant CaMKIIβ clustering even at the lower centrifugation speed. When mixed at equal amounts (0.25 μM each, to a total CaMKII concentration of 0.5 μM used in the other *in vitro* experiments), no significant CaMKIIβ co-clustering was detected (Figures 5E and 5F). However, significant co-clustering of CaMKIIβ was detected when CaMKII concentration was increased with an excess of CaMKIIα (1.5 μM) over CaMKIIβ (0.5 μM), an isoform ratio similar as found in neurons (Figures 5G and 5H). Under these conditions, CaMKIIα still appeared to precipitate more than CaMKIIβ, but this apparent difference was not statistically significant (p = 0.053). When the isoforms were instead co-expressed (in order to allow formation of heteromeric holoenzymes), both isoforms precipitated significantly and to an equal extent, as expected (Figure S5).

**Figure 6.**
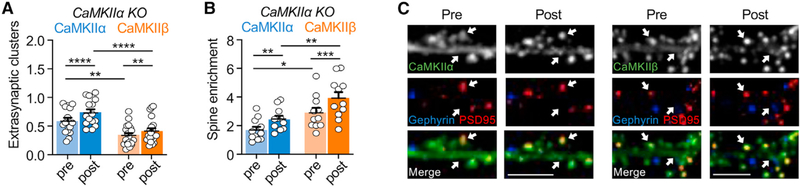
Clustering of CaMKIIα and CaMKIIβ induced by prolonged glutamate in CaMKIIα knockout (KO) neurons Error bars indicate SEM in all panels. **p < 0.01 and ****p<0.0001. Scale bar, 5 μm. (A) Quantification of extra-synaptic clusters induced by excitotoxic glutamate (100 μM glutamate, 5 min) in CaMKIIα KO cultured hippocampal neurons (15–17 DIV) (two-way repeated-measures ANOVA, Bonferroni’s test: ****p < 0.0001 for CaMKIIα pre vs. post, n = 15; **p = 0.0021 for CaMKIIβ pre vs. post, n = 20; **p = 0.0019 for pre CaMKIIα vs. pre CaMKIIβ; ****p < 0.0001 for post CaMKIIα vs. post CaMKIIβ). (B) Quantification of excitatory synapse enrichment induced by excitotoxic glutamate (100 μM glutamate, 5 min) in CaMKIIα KO cultured hippocampal neurons (15–17 DIV) (two-way repeated-measures ANOVA, Bonferroni’s test: **p = 0.0025 for CaMKIIα pre vs. post, n = 11; ****p < 0.0001 for CaMKIIβ pre vs. post, n = 11; *p = 0.0242 for pre CaMKIIα vs. pre CaMKIIβ, **p = 0.0051 for post CaMKIIα vs. post CaMKIIβ). (C) Representative confocal images show overexpressed YFP-labeled CaMKIIα (left) or CaMKIIβ (right), endogenous PSD95 (in red) to mark excitatory synapses, and endogenous gephyrin (in blue) to mark inhibitory synapses.

In order to further test the possibility that CaMKIIβ co-clusters with CaMKIIα in neurons, the clustering experiments were repeated in neurons cultured from CaMKIIα KO mice. In the absence of endogenous CaMKIIα, YFP-CaMKIIβ still clustered both basally and after excitotoxic glutamate insults but to a significantly lesser extent than YFP-CaMKIIα (Figures 6 and S6). Furthermore, the extra-synaptic CaMKIIβ clustering was significantly lower in the CaMKIIα KO neurons compared with WT neurons (Figure S6H). By contrast, in dendritic spines the basal enrichment of CaMKIIβ, but not CaMKIIα, was higher in the CaMKIIα KO neurons compared with WT neurons (see Figures S6E and S6F). This could be an indirect effect of the lower extra-synaptic clustering, or it might be caused more directly by the preferential interaction of CaMKIIβ with F-actin, which is enriched in dendritic spines (Fink et al., 2003; Khan et al., 2016; O’Leary et al., 2006; Okamoto et al., 2007).

Together, our *in vitro* experiments with purified protein and our imaging experiments in neurons show that CaMKIIβ can form clusters also on its own, but to a significantly lesser extent than the CaMKIIα isoform.

## DISCUSSION

**Figure 7.**
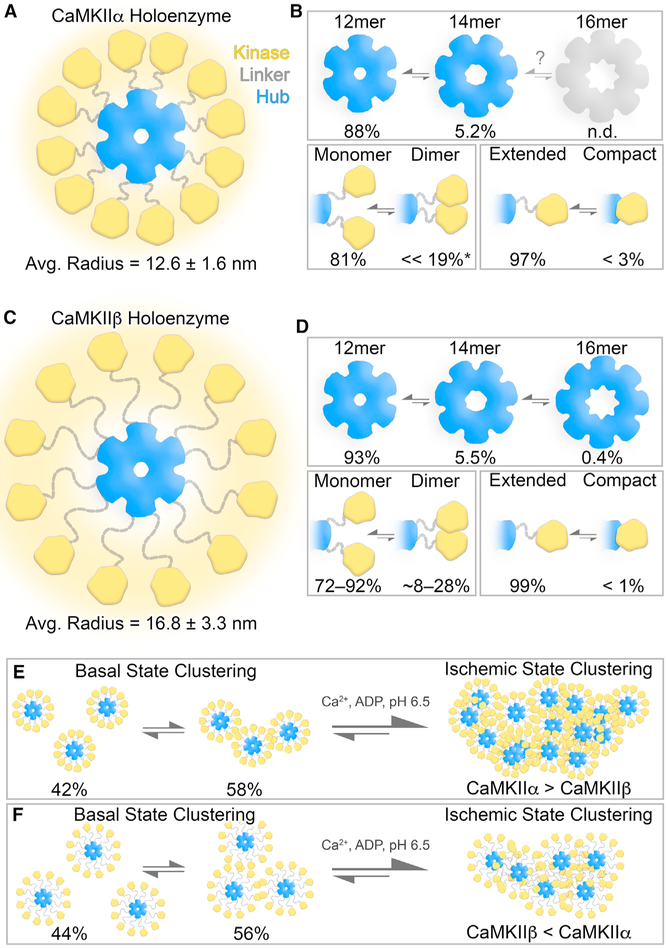
Overview of structural states supported by CaMKII holoenzymes under basal and ischemic conditions (A) Illustration of the 12-meric CaMKIIα holoenzyme under basal conditions, where a flexible linker region (gray) supports a continuum of extended configurations of kinase domains (yellow) with an average radius of 12.6 ± 1.6 nm (SD), from the center of the hub domain (blue). The halo of yellow density represents the variability in kinase domain positioning observed at the single-particle level. (B) Top: oligomeric states of the hub domain resolved by single-particle EM, with the 12-mer most populated (88%), followed by the 14-mer (5.2%); the 16-mer was not detected (n.d.). Bottom left: kinase domains where predominantly resolved as monomers (81%), with a significant population resolved as putative dimers (<<19%*). Asterisk indicates that this value is likely overestimated because of artifacts associated with local crowding in the CaMKIIα isoform (see main text). Bottom right: the majority of kinase domains were resolved in an extended state (97%), while a small subset of subunits were localized within contact distance of the hub domain, consistent with a compact state (<3%). (C) The 12-meric CaMKIIβ holoenzyme, displayed as in (A), supported a significantly larger extension of kinase domain configurations under basal conditions compared with CaMKIIα, with an average radius of 16.8 ± 3.3 nm (SD) facilitated by the longer linker region. (D) Top: CaMKIIβ hub domains were resolved predominantly as 12-mers (93%), followed by 14-mers (5.5%), as well as a 16-meric state (0.4%). Bottom left: kinase domains were also predominantly resolved as monomers (72%–92%), with a significant population resolved as putative dimers (<8%–28%), (bottom right) while the compact state is consistent with only <1% of the population of subunits. (E and F) High-order clustering of holoenzymes detected under basal and ischemic conditions for CaMKIIα (E) and CaMKIIβ (F). Under basal conditions, both isoforms form small clusters or pairs of holoenzymes mediated by kinase domain interactions (58% for CaMKIIα and 56% for CaMKIIβ). Under ischemic conditions inducedin cells or *in vitro*, there is a shift in the equilibrium toward higher-order clusters, where CaMKIIα clustering was found to be significantly greater than for CaMKIIβ

Our comparative structure-function analysis of the CaMKIIα and β holoenzymes revealed notable distinctions between these two major neuronal isoforms, including both expected and unexpected structural differences. Perhaps more important, it also revealed common structural features of CaMKII that are applicable to the regulation of both isoforms. Specifically, these include a highly dynamic activatable-state conformation, the ability to adopt several oligomeric assemblies (mainly 12-mers but also 14- and 16-meric holoenzymes) as well as high-order clusters of holoenzymes, and the detection of kinase domain dimer interactions within CaMKIIβ holoenzymes, a mechanism that could mediate the cooperative activation by CaM for all CaMKII isoforms (see Figure 7). As delineated below, these findings provided both answers and additional questions.

### Holoenzyme expansion and autophosphorylation kinetics

The most obvious difference between the CaMKIIα and β holoenzyme was in their radius of expansion, both in their average radius (12.6 vs. 16.8 nm) and in their maximal radius (16 vs. 23 nm). This difference appears to be also the most predictable one, on the basis of the different lengths of their variable linker regions that tethers the kinase domains to the hub formed by the association domains. On the basis of the average holoenzyme radii, we calculated a local concentration of kinases domains, within the space occupied by a holoenzyme, to be 2 mM for CaMKIIα and 1 mM for β. However, there was no apparent difference in the kinetics of the regulatory T286/T287 autophosphorylation that occurs between the subunits of a holoenzyme. Although this result seemed counterintuitive at first glance, (1) it is predicted by simple Michaelis-Menten kinetics (Dyla and Kjaergaard, 2020), although (2) simple Michaelis-Menten kinetics should not necessarily be expected for this autophosphorylation reaction. When T286 is presented as an exogenous substrate on a peptide, its *K*_m_ is ~10 μM (Coultrap et al., 2010); with this *K*_m_ and with 2 versus 1 mM substrate concentration, Michaelis-Menten kinetics predict reaction speeds of 99.50% versus 99.01% of V_max_ (i.e., a minuscule difference that cannot be resolved in our analyses). However, within the CaMKII holoenzyme, Michaelis-Menten kinetics would break down at least after the first or second autophosphorylation reaction because of the significant substrate depletion. Additionally, there could have been distinct steric positioning of kinase domains in the CaMKIIα versus β holoenzyme due to differences in linker length that could either facilitate or reduce the inter-subunit autophosphorylation. Indeed, the recently described differences in inhibitory autophosphorylation at T305/306 in CaMKIIα versus T306/307 in CaMKIIβ have been attributed to the different linker lengths (Bhattacharyya et al., 2020b). However, whereas T286 autophosphorylation occurs exclusively in *trans* between two subunits of a holoenzyme (Hanson et al., 1994; Rich and Schulman, 1998), the T305/306 autophosphorylation can occur both in *trans* and in *cis* (Colbran, 1993; Cook et al., 2021). Similarly, the different linker lengths in the CaMKIIα versus β isoforms may also affect steric accessibility to external substrate proteins, particularly when the holoenzymes are anchored at postsynaptic protein scaffolds.

### Higher-order assemblies and subcellular CaMKII targeting

A more surprising difference was the reduced propensity of CaMKIIβ to form higher-order clusters under ischemic conditions, both *in vitro* and within neurons. Although it has been previously reported that CaMKIIβ lacks excitotoxicity/ischemia-related clustering (Hudmon et al., 2001), the longer linker in CaMKIIβ should instead have been expected to facilitate this clustering: in the α isoform, clustering is thought to be mediated by kinase domain pairing between holoenzymes (Hudmon et al., 1996; Vest et al., 2009), and a longer linker should facilitate such inter-holoenzyme pairings. The explanation might be that CaMKIIβ can form such pairings, but that the smaller CaMKIIα holoenzymes can pack into larger and/or denser clusters (see Figures 7E and 7F). Indeed, this notion is supported by the preferential detection of CaMKIIβ *in vitro* clustering by high- versus low-speed centrifugation (a fact that may also explain the previous failure to detect these clusters at all). Furthermore, although extra-synaptic CaMKIIβ clustering was significantly less compared with CaMKIIα, a significant level of clustering was observed also for the β isoform, even in CaMKIIα knockout neurons.

Notably, together with the decrease in extra-synaptic clusters, we observed an increase in synaptic CaMKIIβ clusters. This result appears to be in conflict with the notion that the inter-holoenzyme aggregation mediates clustering not only at extra-synaptic sites but also at synapses (Hudmon et al., 2005), a form of subcellular CaMKII movement that is thought to be important in LTP (Barcomb et al., 2016; Barria and Malinow, 2005; Halt et al., 2012; Incontro et al., 2018; Sanhueza et al., 2011). It is well established that CaMKII movement to excitatory synapses requires CaMKII binding to the NMDA receptor subunit GluN2B (Bayer et al., 2001; Buonarati et al., 2020; Halt et al., 2012). However, this does not rule out the possibility that holoenzyme aggregation contributes to this targeting. Furthermore, the reduced propensity of CaMKIIβ to cluster at extra-synaptic sites does not fully rule out the possibility that its clustering could be enhanced at synaptic sites. For instance, the larger holoenzyme radius and less dense clusters of CaMKIIβ might be favorable within the protein-concentrated environment at postsynaptic densities at excitatory synapses. Nonetheless, our results indicate that the higher-order aggregation of CaMKII holoenzymes plays a more important role in cluster formation at extra-synaptic versus synaptic sites.

The function of extra-synaptic CaMKII clustering is still unclear, but it has been proposed to provide neuro-protection by curbing the over-activation of CaMKII after excitotoxicity/ischemia (Aronowski et al., 1992; Hudmon et al., 1996). This mechanism is clearly insufficient to completely prevent the neuronal cell death after excitotoxic/ischemic insults, but CaMKII inhibition can indeed protect neurons in mouse models of stroke or global cerebral ischemia (Deng et al., 2017; Vest et al., 2010). Furthermore, the CaMKII T286A mutation increases clustering (Hudmon et al., 2005; Vest et al., 2009) and decreases neuronal cell death (Deng et al., 2017). However, this correlation allows only limited conclusions, as the T286A mutation also prevents generation of Ca^2+^-independent autonomous CaMKII activity.

### Multivalent interactions within the holoenzymes and cooperative CaMKII regulation

Although kinase domain interactions between holoenzymes are thought to mediate the aggregation of holoenzymes into higher-order assemblies, our results here show direct evidence of kinase domain dimer formation also within the holoenzyme. Previous FRET studies have suggested kinase domain dimers are supported by the CaMKII holoenzyme in cells, but direct detection of dimer formation was not resolved in these studies (Nguyen et al., 2012, 2015; Thaler et al., 2009). Dimer formation was directly observed in a crystal structure of a CaMKII kinases domain (specifically for a *C. elegans* CaMKII that was truncated after the regulatory domain) (Rosenberg et al., 2005). The low affinity of this interaction (*K*_d_ > 100 μM) could be sufficient to support dimer formation on the basis of the 1–2 mM concentration of kinase domains within a holoenzyme. Kinase domain dimerization has also been observed biochemically for isolated kinase domains of all four human isoforms, with similarly low affinity (*K*_d_ values of ~200–600 μM) (Rellos et al., 2010). However, it was entirely unclear if such dimer formation would be possible for kinase domains that are tethered to the central hub of association domains within the holoenzyme. The extended state of CaMKIIβ holoenzymes facilitated the ability to directly resolve kinase domain dimers by EM; although this did not allow detailed resolution of the dimerization surface, the defined bi-lobed densities are consistent with the crystallized CaMKII kinase domain dimer structure.

The putative population of kinase domain dimers for CaMKIIα and β were determined to be ~19% and ~8%, respectively, on the basis of single-particle distance measurements and the average center-to-center distance of ~4.5 nm separating the two subunits in the crystal structure of the kinase domain dimer (see Figure 3E). However, when considering slightly different distance cut-offs of up to 5–6 Å (the difference of approximately one to three pixels in our micrographs), our data suggest dimer population in these two isoforms might be more similar than these reported values, with a likely range of ~8%–30% kinase domain dimers for both isoforms. If CaMKIIα and β holoenzymes contain similar fractions of dimerized kinase domains, both isoforms should show a similar level of cooperative activation by CaM, as was indeed observed here. The dimers are proposed to generate cooperativity, as they are thought to be formed via interactions of the CaM-binding regulatory domains (Rosenberg et al., 2005). Thus, when CaM binds to one subunit, it would disrupt the dimer and thereby also facilitate binding to the other subunit. Although this kinase domain dimerization predicts the observed cooperativity, it also raises some questions. How could a relatively minor fraction of dimers (e.g., ~8%–30%) cause cooperativity with a relatively large Hill coefficient of 1.6?

An additional, or alternative, proposed mechanism for the cooperativity lies in a compact conformation in which a kinase domain folds back onto its own association domain (Chao et al., 2011). A recent elegant cryo-EM study indeed directly demonstrated the existence of this conformation (Sloutsky et al., 2020), whereas our previous and present studies could only set an upper limit to its prevalence (Myers et al., 2017). However, with fewer than 3% of subunits in the compact state, this maximal prevalence is extremely low and consistent to all of the studies (Myers et al., 2017; Sloutsky et al., 2020), which makes it an even less likely candidate mechanism for the observed cooperativity than the kinase domain dimers.

Then, a more likely “neighbor effect” may be that kinase domain dimerization (or disruption of the dimer) affects CaM binding not only to the dimer itself but also to the neighboring subunits. In this way, one single kinase domain dimer could potentiate CaM binding to half of the subunits within a 12-meric CaMKII holoenzyme (i.e., the dimer pair itself plus its four neighbors). A similar model was proposed previously but assumed that the neighbors would also be dimers (Chao et al., 2011); however, with the observed lower occurrence of dimers, the model would have to be modified to include effects also on non-dimerized neighbors. Indeed, such a model is consistent with the emerging view of CaMKII holoenzyme structure, one that is not constrained by a single defined state but rather a highly dynamic conformational ensemble characterized by multiple transient low-affinity interactions (see Figure 7).

An intriguing comparison can be made with other systems hallmarked by multivalent low-affinity interactions organized by intrinsically disordered protein domains that are capable of forming biomolecular condensates (Brangwynne et al., 2009). We suggest that the structural and biophysical properties of the CaMKII holoenzyme structure (e.g., high local concentration of multivalent binding modes) may facilitate the formation of a molecular-scale condensate, at least from a conceptual point of view. Condensates (i.e., liquid-liquid phase separation [LLPS]) have emerged as a regulatory mechanism at synapses (Chen et al., 2020), and the CaMKII interaction with GluN2B has recently been shown to support condensate formation (Hosokawa et al., 2021). What is intriguing about this analogy of individual CaMKII holoenzymes to condensates is that the dissolution of biomolecular condensates is highly cooperative (Banani et al., 2017; Li et al., 2012). Such a model could explain how activation of CaMKII holoenzymes can achieve a higher degree of cooperativity than would be expected on the basis of the extent of kinase domain dimers. Notably, some studies have described Hill coefficients for CaMKII activation by CaM that are even higher than the Hill coefficient of ~1.6 reported here (Chao et al., 2011; Rosenberg et al., 2005). In a model analogous to molecular condensates, each kinase domain would exist in an equilibrium between rapidly exchanging interactions involving multiple neighboring kinase or hub domains, a notion supported by the flexibility of kinase domain positioning with the holoenzymes. The activation of one kinase domain would then disrupt the interaction with multiple neighboring kinase domains, leading to the cooperative collapse or dissolution of the basal state. Although there is currently no direct evidence that a kinase domain dimer (or kinase-hub complex) can affect the positioning of neighboring kinase domains within the holoenzyme, such a proposition appears to be at least more plausible than what can be explained by any single defined state of CaMKII.

### Beyond the 12-mer: Outlook for future studies

The oligomeric state of CaMKII holoenzyme is clearly important for facilitating its physiological roles in Ca^2+^ frequency detection and in regulating LTP and LDP (Coultrap et al., 2014; De Koninck and Schulman, 1998; Giese et al., 1998; Hanson et al., 1994). The predominant state of both CaMKIIα and β holoenzymes is the 12-meric assembly (see Figure 2 and Myers et al., 2017). However, both holoenzymes can also support 14-meric assemblies, and here we show that CaMKIIβ can even support a 16-meric assembly. Such a high oligomeric state of the CaMKII hub domain appears unique in metazoans. Interestingly, bacteria and algae species contain orphan proteins with sequence and structural homology to CaMKII hub domains that adopt 16- to 20-mers (McSpadden et al., 2019). Crystallographic analysis of the hub-like assembly from *Chlamydomonas reinhardtii* revealed an 18-meric structure with striking similarity to CaMKII hub assemblies but with increased hydrogen bonding at the lateral subunit interface. Remarkably, when these hydrogen bonding residues were incorporated into the CaMKIIα hub domain, it assembled as 14- and 16-mers (McSpadden et al., 2019). It is currently unclear if wild-type CaMKIIα holoenzymes support a 16-meric assembly, but if they can, it is likely a very minor population, as we have found that this state only represents ~0.4% of the population in CaMKIIβ holoenzymes under basal conditions, and both isoforms lack the hydrogen-bonding potential identified in the algae hub-like assembly.

These observations raise the important question as to what is the functional significance of higher-order oligomeric states of the CaMKII holoenzyme. It has recently been shown that under activating conditions, CaMKII subunits (dimeric pairs) are capable of undergoing exchange between other activated or non-activated holoenzymes (Bhattacharyya et al., 2016; Stratton et al., 2014). Although subunit exchange was minimal under basal conditions, it is possible that the small population of 14-mers/16-mers represented high-energy intermediate states involved in the subunit exchange mechanism. Activation of the CaMKII holoenzyme is thought to destabilize the hub complex, through interactions with the regulatory domain (Bhattacharyya et al., 2016; Karandur et al., 2020; Stratton et al., 2014). Thus, the 14-mer and 16-mer states could also provide a storage mechanism for pools of potentiated subunits to be released under activating conditions. Intriguingly, this subunit exchange mechanism has been proposed to enable the propagation of CaMKII activation and other neuronal plasticity mechanisms that could play important roles in learning and memory (Bayer and Schulman, 2019; Bhattacharyya et al., 2020a). Future studies will be needed to further test these hypotheses and to shed light on the mechanistic basis for how such regulatory functions are achieved.

### Limitations of the study

In addition to the technical and conceptual limitations described above, it should be noted that the observations of structural behavior of CaMKII holoenzymes by EM are made under dilute *in vitro* conditions. Although significant effort was made to correlate these behaviors to functional and/or structural phenomena inside cells, there are many factors within a native cellular environment (e.g., temperature, molecular crowding, interactions with cognate binding partners) that may augment the structural features and/or dynamic properties of CaMKII holoenzymes that are described in this work. Future studies will be targeted at defining how these cellular conditions contribute to the molecular plasticity and functional properties of CaMKII.

## STAR★METHODS

### RESOURCE AVAILABILITY

#### Lead contact

Further information and requests for resources and reagents should be directed to and will be fulfilled by the Lead Contact, Steve L. Reichow (reichow@pdx.edu).

#### Materials availability

This study did not generate new unique reagents.

#### Data and code availability

- Raw and analyzed data contributing to this work have been deposited at Mendeley and are publicly available as of the date of publication. The DOI is listed in the key resources table. Microscopy data reported in this paper will be shared by the lead contact upon request.
- This study does not report original code.
- Any additional information required to reanalyze the data reported in this paper is available from the lead contact upon request.

### EXPERIMENTAL MODEL AND SUBJECT DETAILS

#### Hippocampal cultured neurons

Mixed sex pups from homozygous mice (P1–2; on a C57BL/6 background) were used to prepare dissociated hippocampal cultures for live imaging. The CaMKII KO mice were described previously (Coultrap et al., 2014). To prepare primary hippocampal neurons from WT or mutant mice, hippocampi were dissected from mixed sex mouse pups (P1–2), dissociated in papain for 30 min, and plated at 200–300,000 cells/mL for imaging. At DIV 14–15, neurons were transfected with 1 μg total cDNA per well using Lipofectamine 2000 (Invitrogen). At DIV 16–17, neurons were treated and imaged. All animal procedures were approved by the Institutional Animal Care and Use Committee (IACUC) of the University of Colorado Anschutz Medical Campus and were carried out in accordance with NIH best practices for animal use. All animals were housed in ventilated cages on a 12 h light/ 12 h dark cycle and were provided ad libitum access to food and water.

### METHOD DETAILS

#### DNA constructs

CaMKIIα and β constructs are based on a pcDNA3 backbone (Addgene #13033). For imaging studies, the YFP-CaMKII constructs contained N-terminal fusion to an EYFP with its A206 mutated to reduce dimerization of the YFP, as described previously (Bayer et al., 2006; Zacharias et al., 2002). For the biochemical self-association studies with co-expressed isoforms, the YFP tag was removed. All CaMKIIα and β used here were the respective major splice variants in adult mammalian brain of each isoform, i.e. β, not βB; and β, not β’, βe, βe’, or βM etc. (Cook et al., 2018). Expression vectors for the GFP-labeled FingR intrabodies targeting PSD-95 and gephyrin were kindly provided by Dr. Donald Arnold (University of Southern California, Los Angeles, CA, USA) as previously characterized (Gross et al., 2013; Mora et al., 2013). The fluorophore label was exchanged using Gibson Assembly to contain the following tags in place of GFP: PSD-95-FingR-mTurquois and gephyrin-FingR-mCherry.

#### CaMKII and CaM purification

For biochemistry and electron microscopy, homomeric rodent CaMKIIα and β holoenzymes were expressed in eukaryotic *Sf9* cells via baculovirus and purified using a phosphor-cellulose column followed by a CaM-sepharose column; CaM was purified after expression in BL21 bacteria (see also Coultrap and Bayer, 2012a; Singla et al., 2001). The heteromeric CaMKII used in Figure S5 was generated by co-expression in HEK293 cells and purified using the CaM-sepharose step (Coultrap and Bayer, 2012a).

#### Electron microscopy

Full length purified CaMKIIβ holoenzymes were prepared for negative stain EM by diluting a freshly thawed aliquot of protein (1:40 vol vol^−1^) in EM buffer containing 50 mM HEPES (pH 7.4), 120 mM KCl and 0.5 mM EGTA. A 3 μL drop of the diluted specimen (~0.35 μM holoenzyme concentration) was applied to a glow-discharged continuous carbon coated EM grid (Ted Pella). Excess protein was removed by blotting with filter paper and washing twice with EM buffer. The specimen was then stained with freshly prepared (0.75% wt vol^−1^) uranyl formate (SPI-Chem), blotted and dried with laminar air flow.

Negatively stained specimens were visualized on a 120kV TEM (Tecnai iCorr, FEI) and digital micrographs were manually collected on a 2K x 2K CCD camera (Eagle, FEI) at a nominal magnification of 49,000 × at the specimen level. Micrographs were collected with a calibrated pixel size of 4.37 Å and defocus of 1.5 – 2.5 μm. A total of 907 micrographs were collected and screened for astigmatism and drift based on Thon rings in the power spectra after determination of contrast transfer function (CTF) parameters in EMAN2.1 (Tang et al., 2007). 17,347 single particles images were manually picked in EMAN 2.1 and extracted with a box size of 144 × 144 pixels. Boxed images used for single particle analysis were of isolated holoenzymes that were not associated with neighboring holoenzymes (such as the particles examined for clustering). Reference-free 2D classification and variance analysis was conducted in RELION3.0b (Zivanov et al., 2018) on CTF-corrected (phase-flipped), using various masking strategies: holoenzyme = 55.0 nm mask, hub domain = 15.0 nm mask, and kinase domains = inner mask 13.5 nm, without applied symmetry. For comparative analysis, the previously acquired negative stain EM dataset of 10,902 untilted CaMKIIα single-particle images (Myers et al., 2017) was reprocessed in EMAN 2.1 and RELION3.0b, as described above using a box size of 128 × 128 pixels.

#### Single particle measurements and statistical analysis

Statistical analyses of individual holoenzyme particle dimensions were obtained from particle lengths using the measurement tool in EMAN2.1, as previously described (Myers et al., 2017). A radius of extension for individual kinase subunits (*n* = 926 measurements obtained from 95 holoenzymes) was determined by measuring the distance from the center of the pore in the hub domain complex to the center of each peripheral density corresponding to the kinase domains. For each of these measurements, a distance of 2.25 nm was appended (corresponding to the average radius of the kinase domain) to yield a value that represents the full extension of the kinase domain. Inter-molecular kinase separation was determined by measuring from the center of one peripheral kinase density to the center of the nearest clockwise neighboring kinase density. For the linker extension analysis, the hub average radius (5.5 nm) and the kinase average radius (2.25 nm) was subtracted from the raw hub to kinase radius measurements, as a representative distance of linker extension and comparison to random chain polymer models (traditional random walk model) (Flory, 1975). The local concentration of kinase domains in the context of a single dodecameric holoenzyme was determined by assuming a spherical volume with the radius corresponding to the average radius extension for each isoform ± 2 x standard deviation appended. To analyze intermolecular clustering of holoenzymes, a random subset of raw micrographs was visually inspected for CaMKIIα (1207 particles) and CaMKIIβ (1255 particles) by counting the total number of holoenzymes and the number of non-clustering holoenzymes (designated as being separated by at least ~1.5 times the particle diameter from a nearest neighbor). For statistical comparisons, an F-test was performed to determine the difference in sample variances, followed by a T-test (two sample assuming unequal variances). All statistical analysis and graphical interpretations were done using libraries in python 3.

#### Pseudo-atomic modeling of the CaMKII 16-meric hub assembly and back-projection analysis

A pseudo-atomic model of the hexadecameric (16-mer) CaMKII hub assembly was built manually, using the atomic coordinates corresponding to a vertical hub dimer extracted from the previously published dodecameric assembly (PDB 5IG3) (Bhattacharyya et al., 2016), with an applied C8 symmetry (overall D8 symmetry) that was adjusted to approximately fit within the experimental 2D class average density following C8 symmetry averaging (outer diameter of ~13 nm) and to obtain minimal steric overlap between neighboring hub domains. For comparing with 2D class averages, hub models where filtered to 3.0 nm and 2D back projections were generated in RELION (Scheres, 2012).

#### Live imaging of hippocampal cultured neurons

All images were acquired using an Axio Observer microscope (Carl Zeiss) fitted with a 63x Plan-Apo/1.4 numerical aperture objective, using 445, 515, 561, and 647 nm laser excitation and a CSU-XI spinning disk confocal scan head (Yokogawa) coupled to an Evolve 512 EM-CCD camera (Photometrics). During image acquisition, neurons were maintained at 34°C in 10 mM HEPES-buffered neuronal media. After baseline imaging (‘pre’), cells were treated with 100 μM glutamate and imaged 5 min later (‘post’). Tertiary dendrites from pyramidal spiny neurons were selected from maximum intensity projections of confocal Z stacks. Slidebook 6.0 software (Intelligent Imaging Innovations [3i]) was used to analyze CaMKII-YFP at excitatory (PSD-95) and inhibitory (gephyrin) synapses. Specifically, the mean YFP intensity at PSD-95 or gephyrin threshold masks on a given dendrite was divided by the mean YFP intensity of a line drawn in the adjacent dendritic shaft. ImageJ (National Institute of Health) was used to analyze CaMKII at extra-synaptic sites. Specifically, the thresholded mask for PSD-95 puncta was subtracted from the CaMKII channel and the remaining CaMKII clusters (0.1 μm < cluster<1.0 μm) were quantified as number per 10 μm dendrite.

#### CaMKII *in vitro* reactions

CaMKII self-associations assays were performed similar to previous work (Vest et al., 2009). Purified CaMKII (0.5 μM or as indicated) was pre-cleared by ultracentrifugation (100,000 × g) at 4°C for 45 min, then combined with 25 mM PIPES pH 6.4, 20 mM KCl, 10 mM MgCl_2_, 0.1 mg/mL bovine serum albumin, 0.1% Tween 20, 0.5 mM dithiothreitol, 2 mM CaCl_2_,1 μM CaM, and 1 mM ADP. Control samples were instead combined with 25 mM PIPES pH 7.4, 20 mM KCl, 10 mM MgCl_2_, 0.1 mg/mL bovine serum albumin, 0.1% Tween 20, 0.5 mM dithiothreitol, and 50 mM EGTA. The mixtures were prepared on ice and then incubated for 5 min at room temperature prior to centrifugation (16,000 × g) or ultracentrifugation (100,000 × g) at 4°C for 30 min. CaMKII in the supernatant and pellet was detected via immunoblot.

For autophosphorylation assays, purified CaMKII (0.1 uM) was pre-cleared by ultracentrifugation (100,000 × g) at 4°C for 45 min, then combined with 25 mM PIPES pH 7.1, 10 mM MgCl_2_, 0.1 mg/mL bovine serum albumin, 4 mM CaCl_2_,3 μM CaM, and 1 mM ATP. The mixtures were prepared on ice and then incubated at 30°C for 0 sec, 30 sec, 180 sec, or 15 min. Autophosphorylation at pT286-CaMKIIα and pT287-CaMKIIβ was detected via immunoblot.

For kinase activity assays, purified CaMKII (2.5 nM) was combined with 50 mM PIPES pH 7.1, 10 mM MgCl_2_, 0.1 mg/mL bovine serum albumin, 1 mM CaCl_2_, 100 μM [γ−^32^P] ATP, 75 μM syntide-2 substrate peptide, and 0.6 μM to 6 μM CaM. The mixtures were prepared on ice and then pre-incubated at 30°C for 5 min. CaMKII was added and the reaction was allowed to progress for 1 min at 30°C. To stop the reaction, 40 μL of the 50 μL reaction mixture was spotted onto P81 cation exchange chromatography paper (Whatman) squares. After extensive washes with water, phosphorylation of the substrate peptide bound to the P81 paper was measured by liquid scintillation counting. CaMKII activity (Vm; reactions/kinase/min) was quantified as a fraction of maximal activity (Vmax) for each experiment. Data were fitted using a non-linear regression with variable slope.

#### SDS-PAGE and immunoblot

Protein content was determined using the Pierce BCA protein assay (Thermo Fisher). 4–10 μg of total protein in Laemmli sample buffer was resolved by SDS-PAGE on 9% polyacrylamide gels and transferred to polyvinylidene fluoride (PVDF) membrane at 24 V for 1–2 h at 4°C in transfer buffer containing: 12% MeOH, 25 mM Tris, and 192 mM glycine. All membranes were blocked in 5% nonfat dried milk in TBS-T (20 mM Tris pH 7.4, 150 mM NaCl, 0.1% Tween 20) before primary antibody incubation for 2 h at room temperature or overnight at 4°C. Antibodies and dilutions were as follows: rabbit anti-CaMKII (Genetex; 1:1000), mouse anti-CaMKIIα (in house CBa2; 1:1000), mouse anti- CaMKIIβ (in house CBb1; 1:1000), and anti-pT286 (Phospho-Solutions; 1:2000). Blots were then washed in TBS-T, incubated in horseradish peroxidase-labeled goat anti-rabbit or goat anti-mouse antibodies (GE Healthcare; 1:10,000) for 1 h at room temperature, and washed again in TBS-T. Immunoreactive signal was visualized by chemiluminescence (Super Signal West Femto, Thermo Fisher) using the Chemi-Imager 4400 system (Alpha-Innotech). Densitometry analysis was performed using ImageJ (National Institute of Health), with all samples normalized to control conditions on the same gel.

### QUANTIFICATION AND STATISTICAL ANALYSIS

Structural measurement data are shown as mean ± SEM, with standard deviation or 95% confidence interval were indicated using Microsoft Excel or SciPy. Comparisons between isoforms for kinase radius and separation measurements were done with a f-test to determine sample variance differences, followed by a t-test (two sampled assuming unequal variance). Functional data are shown as mean ± SEM and analyzed using Prism (GraphPad) software. Comparisons between pre- and post-glutamate images in neurons were analyzed using repeated measures two-way ANOVA with Bonferroni’s post-hoc test. Self-association assays with purified CaMKII were analyzed using two-way ANOVA with Bonferroni’s post-hoc test. Kinase activity assays to assess CaM dose/response were analyzed using extra-sum-of-squares F-test. Comparisons in WT neurons at inhibitory synapses were analyzed using paired, two-tailed Student’s t-test. Statistical significance and sample size (n) are indicated in the figure legends.

## Supplementary Material

Supplement

## Figures and Tables

**KEY RESOURCES TABLE:** 

REAGENT or RESOURCE	SOURCE	IDENTIFIER

Antibodies		

CaMKII pan	Genetex	GTX127939; RRID: AB_2492051
CaMKIIα	Made in house	CBα2; RRID: AB_2533032
CaMKIIβ	Made in house	CBβ1; RRID: AB_2533045
pT286	Phospho-Solutions	p1005–286; RRID: AB_2492051
Rabbit	GE Healthcare	NA934V; RRID: AB_772206
Mouse	GE Healthcare	NA931V; RRID: AB_772210

Chemicals, peptides, and recombinant proteins		

Glutamate	Sigma	6106-04-3
Papain	Worthington	LS 03126
Lipofectamine 2000	Invitrogen	11668027
B-27 supplement	GIBCO	17504044
complete protease inhibitor cocktail	Roche	1187380001
Microcystin-LR	Calbiochem	475815
Uranyl formate	SPI-Chem	16984-59-1
Sodium hydroxide	Fisher Scientific	1310-73-2,497-19-8
Calmodulin	Made in house	CaM
Ca^2+^/CaM-dependent kinase II α	Made in house	CaMKII α
Ca^2+^/CaM-dependent kinase II β	Made in house	CaMKIIβ

Critical commercial assays		

Pierce BCA protein assay	Thermo Fisher	23225
SuperSignal West Femto	Thermo Fisher	34095

Deposited data		

Raw and analyzed data	This paper	Mendeley Data, V1, https://doi.org/10.17632/35gf4sjxmb.1

Experimental models: Organisms/strains		

Rat: Sprague-Dawley	Charles River Laboratory	N/A
Mouse: wild type: C57BL/6	Charles River Laboratory	N/A
Mouse: CaMKIIα KO: C57BL/6	(Coultrap et al., 2014)	N/A

Recombinant DNA		

PSD-95-FingR-GFP	(Gross et al., 2013)	RRID: Addgene_46295
Gephyrin-FingR-GFP	(Gross et al., 2013)	RRID: Addgene_46296
YFP-CaMKIIα	(Bayer et al., 2006; Shen and Meyer, 1999)	N/A
YFP-CaMKIIβ	Made in house	N/A

Software and algorithms		

Slidebook 6.0	Intelligent Imaging Innovations (3i)	RRID:SCR_014300
Prism 7.0	Graphpad	RRID: SCR_002798
AlphaEase FC 4.0	Alpha Innotech	N/A
ImageJ	NIH	RRID:SCR_003070
EMAN2.1	NIH	RRID:SCR_018867
RELION3.0b	MRC (United Kingdom)	RRID:SCR_016274
UCSF Chimera	NIH	RRID:SCR_004097
Python 3	Python Software Foundation	RRID:SCR_008394
Microsoft Excel	Microsoft	RRID:SCR_016137

Other		

400 mesh, 3.0 mm O.D. copper grid	PELCO	1GC400

## Highlights

- CaMKIIβ holoenzymes assemble mainly 12-mers but also 14-mers and 16-mers
- CaMKIIβ holoenzymes have an extended radius in comparison with CaMKIIα
- CaMKIIβ has lower propensity for inter-holoenzyme aggregation
- Kinase domain dimers resolved within CaMKII holoenzymes enable cooperativity
