# Supplementary material for: Conserved and divergent features of neuronal CaMKII holoenzyme structure, function, and high-order assembly
Source: Cell Rep. Author manuscript; Available in PMC 2022 Apr 6. (PMC8985225; doi:10.1016/j.celrep.2021.110168)
Supplement: Supplement [file NIHMS1792130-supplement-Supplement.pdf]

SUPPLEMENTAL FIGURES AND LEGENDS

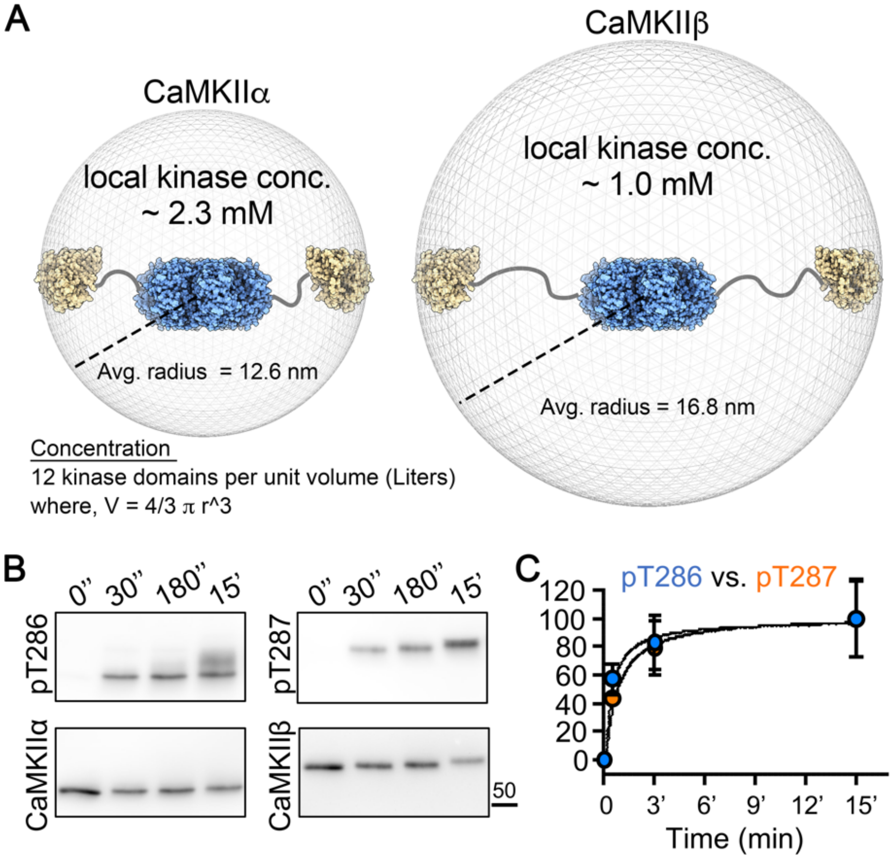

Figure S1. Effect of local kinase domain concentration on pT286-CaMKIIα and pT287-CaMKIIβ in vitro kinetics. Related to Figure 1.

Quantifications show mean ± SEM.

(A) Estimated local kinase concentration in CaMKIIα (*left*) and CaMKIIβ (*right*) holoenzymes. Calculations assume individual kinase domains (12 per holoenzyme) may occupy a spherical volume that is approximated by the average radius of the holoenzyme complex.

(B) Representative immunoblots showing autophosphorylated T286-CaMKIIα (*left*) and T287-CaMKIIβ (*right*) after 0 sec, 30 sec, 180 sec, and 15 min at 30° C. Reactions were performed with 100 nM purified kinase in buffered solution containing 2 mM Ca^{2+}, 3 µM CaM, 10 mM Mg^{2+} and 1 mM ATP.

(C) Quantified time-course of normalized pT286-CaMKIIα versus pT287-CaMKIIβ (n=4). Solid lines through the data points represent nonlinear best-fits.

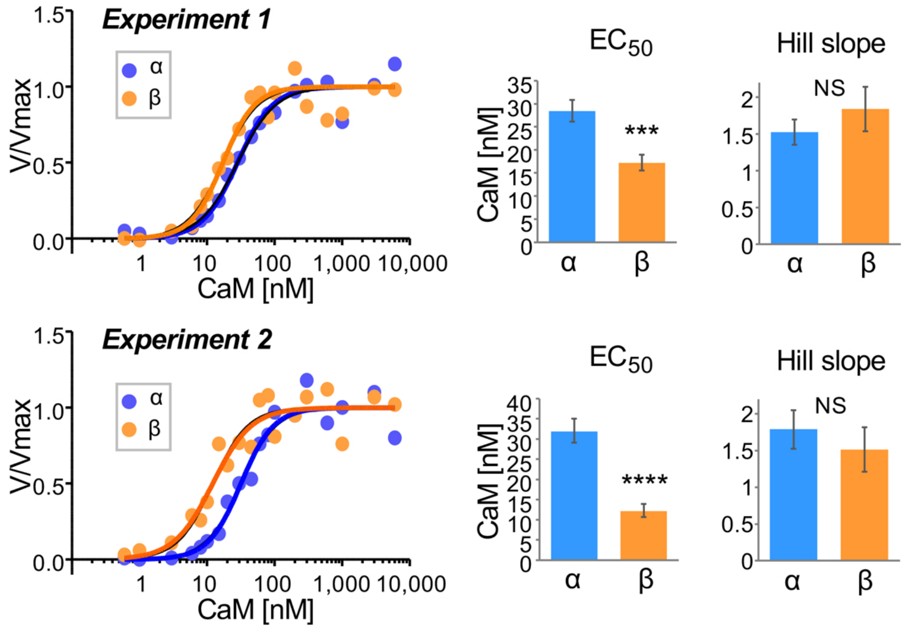

Figure S2. CaMKIIα and CaMKIIβ activation by CaM: individual experiments. Related to Figure 3.

Quantifications show mean ± SEM. ***p<0.001, ****p<0.0001.

In vitro CaMKII activity in response to varying Ca^{2+}/CaM (0.6 nM to 6 µM CaM). The two curve fits shown are based on data from independent reactions: experiment 1 (*top*) and experiment 2 (*bottom*). Both experiments showed a reduced EC_{50} for CaMKIIβ compared to α (extra-sum-of-squares F-test, ***p=0.006 for experiment 1 and ****p<0.0001 for experiment 2). However, both experiments demonstrated a Hill slope >1 for CaMKII, with no differences detected between α and β isoforms (extra-sum-of-squares F-test, p=0.3619 for experiment 1 and p=0.4921 for experiment 2).

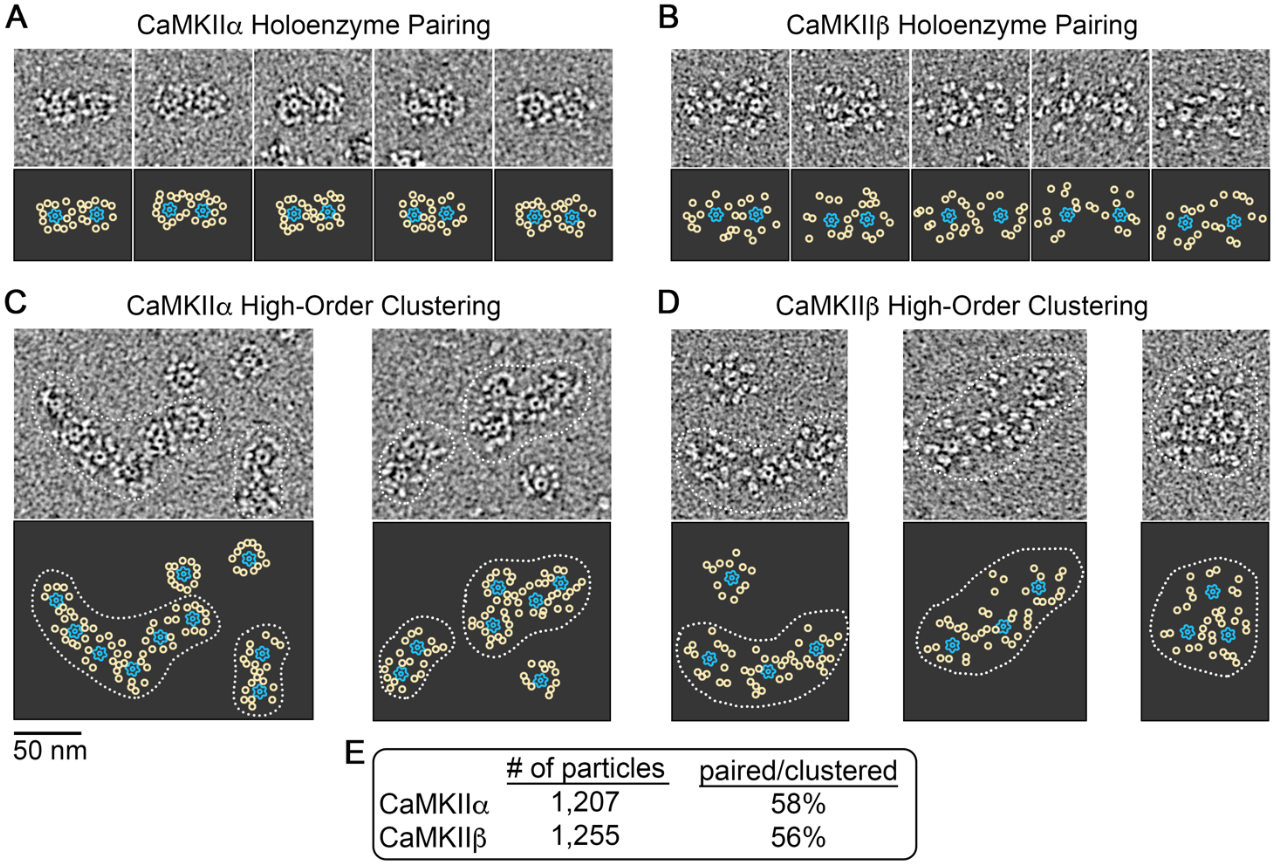

E

	# of particles	paired/clustered
CaMKIIα	1,207	58%
CaMKIIβ	1,255	56%

Figure S3. EM analysis of CaMKII holoenzyme clustering under basal conditions. Related to Figure 4.

(A and B) *Top,* representative EM images showing apparent holoenzyme paring for CaMKIIα and CaMKIIβ, respectively. *Bottom*, displays annotated representation of resolved holoenzyme domains, with the hub domain represented as blue outline and kinase domains as yellow circles.

(C and D) *Top,* representative EM images showing apparent high-order clustering of holoenzymes (3 or more) for CaMKIIα and CaMKIIβ, respectively (dotted outlines). *Bottom*, annotated representation of resolved holoenzyme domains, as in panels (A) and (B), with holoenzyme clusters indicated by dotted outline. Scale bar = 50 nm in panels (A – D).

(E) Percentage of paired/clustered holoenzymes as assigned by visual inspection of micrographs for CaMKIIα and CaMKIIβ image datasets (n=1,207 particles for CaMKIIα and 1,255 particles for CaMKIIβ), indicate an approximately equal propensity for holoenzyme clustering between these two isoforms under basal-state conditions.

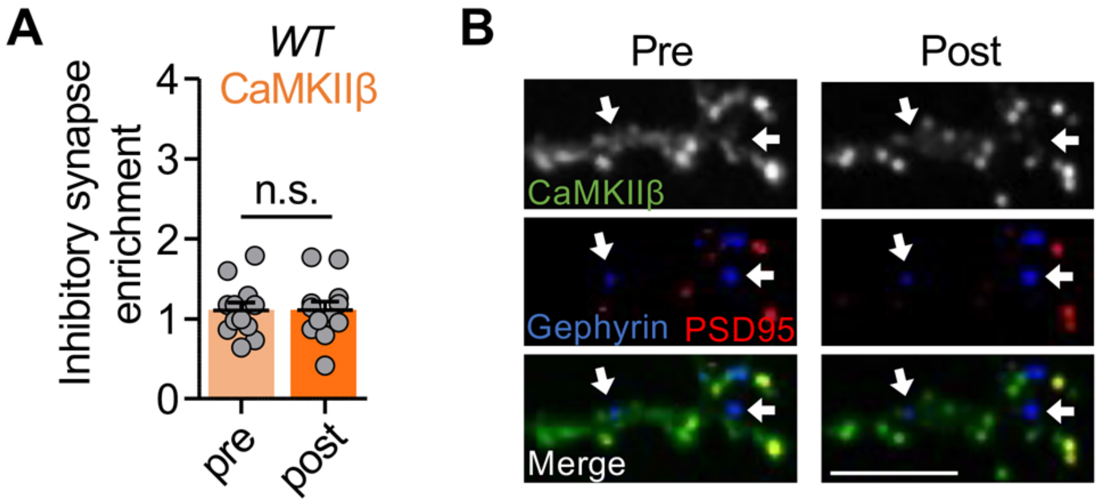

Figure S4. CaMKIIβ does not move to inhibitory synapses following prolonged glutamate. Related to Figure 4.

Quantifications show mean ± SEM. Scale bar, 5 µm.

(A) Quantification of inhibitory synapse enrichment induced by excitotoxic glutamate (100 µM glutamate, 5 min) in WT cultured hippocampal neurons (paired two-tailed t-test: $p=0.4689$).

(B) Representative confocal images show overexpressed CaMKIIβ, endogenous PSD95 (in red) to mark excitatory synapses, and endogenous gephyrin (in blue) to mark inhibitory synapses.

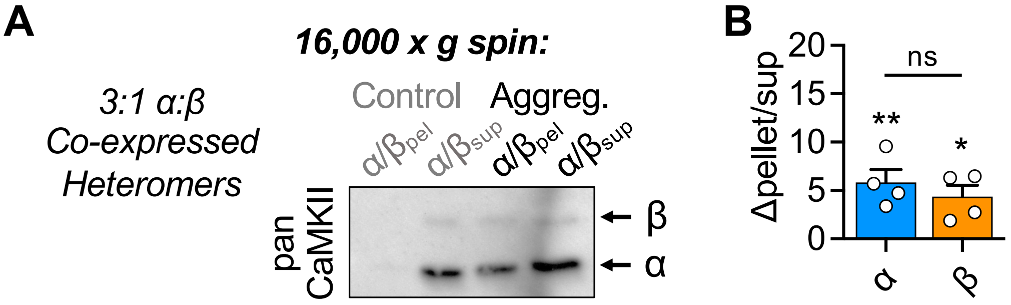

Figure S5. Aggregation in heteromeric CaMKIIα/β holoenzymes. Related to Figure 5.
Quantifications show mean ± SEM. ***p<0.001.

Aggregation was induced by 2 mM Ca^{2+}, 1 µM CaM, and 1 mM ADP at low pH (6.4) for 5 min at room-temperature. Control samples were incubated in 50 mM EGTA at pH 7.4 and normalized to 1.

(A) Representative immunoblot for CaMKIIα/β heteromers. CaMKIIα and CaMKIIβ were co-expressed in HEK-293 cells at 3:1 α:β, then homogenized and purified via CaM-sepharose. Aggregates were detected in 16,000x*g* pellets.

(B) Quantification of change in pellet enrichment. Both CaMKIIα and CaMKIIβ showed significant clustering under aggregation conditions in 3:1 co-expressed heteromers, compared to control (two-way ANOVA, Bonferroni's test: **p=0.0047 for CaMKIIα; *p=0.0416 for CaMKIIβ). No significant differences in self-aggregation levels were detected between CaMKIIα and CaMKIIβ (two-way ANOVA, Bonferroni's test, n.s.: p=0.2266).

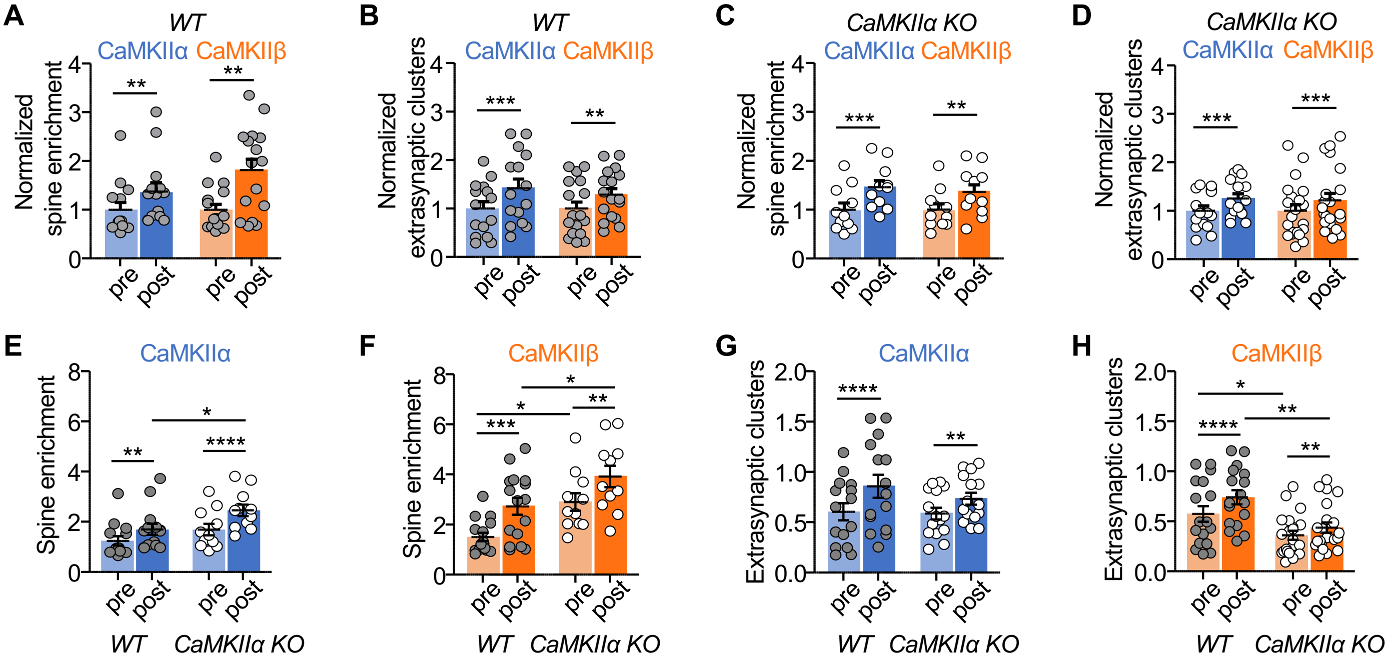

Figure S6. CaMKII movement normalized and compared by genotype. Related to Figures 4 and 6.

Panels A-D show CaMKIIα vs. CaMKIIβ isoform comparison after normalizing to baseline. Panels E-H show WT vs. CaMKIIα KO genotype comparison.

Quantifications show mean ± SEM. *p<0.05, **p<0.01, ***p<0.001, ****p<0.0001.

(A) CaMKIIα vs. CaMKIIβ movement to excitatory synapses in WT (two-way repeated-measures ANOVA, Bonferroni's test: **p=0.0012 for pre vs. post in CaMKIIα; **p=0.0017 for pre vs. post in CaMKIIβ).

(B) CaMKIIα vs. CaMKIIβ clustering at extra-synaptic cluster sites in WT (two-way repeated-measures ANOVA, Bonferroni's test: ***p=0.0001 for pre vs. post in CaMKIIα; **p=0.0035 for pre vs. post in CaMKIIβ).

(C) CaMKIIα vs. CaMKIIβ movement to excitatory synapses in CaMKIIα KO (two-way repeated-measures ANOVA, Bonferroni's test: ***p=0.0001 for pre vs. post in CaMKIIα; **p=0.0017 for pre vs. post in CaMKIIβ).

(D) CaMKIIα vs. CaMKIIβ clustering at extra-synaptic cluster sites in CaMKIIα KO (two-way repeated-measures ANOVA, Bonferroni's test: **p=0.0004 for pre vs. post in CaMKIIα; **p=0.0007 for pre vs. post in CaMKIIβ).

(E) CaMKIIα movement to excitatory synapses in WT vs. CaMKIIα KO (two-way repeated-measures ANOVA, Bonferroni's test: **p=0.0018 for pre vs. post in WT; ****p<0.0001 for pre vs. post in CaMKIIα KO; *p=0.0344 for post WT vs. post CaMKIIα KO).

(F) CaMKIIβ movement to excitatory synapses in WT vs. CaMKIIα KO (two-way repeated-measures ANOVA, Bonferroni's test: ****p<0.0001 for pre vs. post in WT; **p=0.0024 for pre vs. post in CaMKIIα KO; *p=0.0230 for pre WT vs. pre CaMKIIα KO; *p=0.0078 for post WT vs. post CaMKIIα KO).

(G) CaMKIIα extra-synaptic clustering in WT vs. CaMKIIα KO (two-way repeated-measures ANOVA, Bonferroni's test: ****p<0.0001 for pre vs. post in WT; *p=0.0179 for pre vs. post in CaMKIIα KO).

(H) CaMKIIβ extra-synaptic clustering in WT vs. CaMKIIα KO (two-way repeated-measures ANOVA, Bonferroni's test: ****p<0.0001 for pre vs. post in WT; **p=0.0058 for pre vs. post in CaMKIIα KO; *p=0.0303 for pre WT vs. pre CaMKIIα KO; **p=0.0015 for post WT vs. post CaMKIIα KO).
